# Halogenated Dibenzo[*f*,*h*]quinoxaline Units Constructed 2D‐Conjugated Guest Acceptors for 19% Efficiency Organic Solar Cells

**DOI:** 10.1002/advs.202403334

**Published:** 2024-06-17

**Authors:** Jingshun Gao, Hairui Bai, Ping Li, Yibo Zhou, Wenyan Su, Chang Liu, Xiaoxiao Li, Yue Wu, Bin Hu, Zezhou Liang, Zhaozhao Bi, Xiong Li, Lihe Yan, Huiling Du, Guanghao Lu, Chao Gao, Kun Wang, Yuhang Liu, Wei Ma, Qunping Fan

**Affiliations:** ^1^ State Key Laboratory for Mechanical Behavior of Materials Xi'an Jiaotong University Xi'an 710049 China; ^2^ School of Materials and Chemical Engineering Zhongyuan University of Technology Zhengzhou 451191 China; ^3^ School of Materials Science and Engineering Xi'an University of Science and Technology Xi'an 710054 China; ^4^ Laboratory of Advanced Optoelectronic Materials Suzhou Key Laboratory of Novel Semiconductor‐Optoelectronics Materials and Devices College of Chemistry Chemical Engineering and Materials Science Soochow University Suzhou Jiangsu 215123 China; ^5^ Frontier Institute of Science and Technology Xi'an Jiaotong University Xi'an 710054 China; ^6^ Key Laboratory for Physical Electronics and Devices of the Ministry of Education & Shaanxi Key Lab of Photonic Technique for Information School of Electronics Science & Engineering Faculty of Electronic and Information Engineering Xi'an Jiaotong University Xi'an 710049 China; ^7^ Department of Physics Beijing Technology and Business University Beijing 100048 China; ^8^ Key Laboratory of Liquid Crystal and Organic Photovoltaic Materials State Key Laboratory of Fluorine & Nitrogen Chemicals Xi'an Modern Chemistry Research Institute Xi'an 710065 China

**Keywords:** 2D‐conjugation, dibenzo[f,h]quinoxaline, halogenation, organic solar cells, power conversion efficiency

## Abstract

Halogenation of Y‐series small‐molecule acceptors (Y‐SMAs) is identified as an effective strategy to optimize photoelectric properties for achieving improved power‐conversion‐efficiencies (PCEs) in binary organic solar cells (OSCs). However, the effect of different halogenation in the 2D‐structured large π‐fused core of guest Y‐SMAs on ternary OSCs has not yet been systematically studied. Herein, four 2D‐conjugated Y‐SMAs (X‐QTP‐4F, including halogen‐free H‐QTP‐4F, chlorinated Cl‐QTP‐4F, brominated Br‐QTP‐4F, and iodinated I‐QTP‐4F) by attaching different halogens into 2D‐conjugation extended dibenzo[*f*,*h*]quinoxaline core are developed. Among these X‐QTP‐4F, Cl‐QTP‐4F has a higher absorption coefficient, optimized molecular crystallinity and packing, suitable cascade energy levels, and complementary absorption with PM6:L8‐BO host. Moreover, among ternary PM6:L8‐BO:X‐QTP‐4F blends, PM6:L8‐BO:Cl‐QTP‐4F obtains a more uniform and size‐suitable fibrillary network morphology, improved molecular crystallinity and packing, as well as optimized vertical phase distribution, thus boosting charge generation, transport, extraction, and suppressing energy loss of OSCs. Consequently, the PM6:L8‐BO:Cl‐QTP‐4F‐based OSCs achieve a 19.0% efficiency, which is among the state‐of‐the‐art OSCs based on 2D‐conjugated Y‐SMAs and superior to these devices based on PM6:L8‐BO host (17.70%) and with guests of H‐QTP‐4F (18.23%), Br‐QTP‐4F (18.39%), and I‐QTP‐4F (17.62%). The work indicates that halogenation in 2D‐structured dibenzo[*f*,*h*]quinoxaline core of Y‐SMAs guests is a promising strategy to gain efficient ternary OSCs.

## Graphical abstract

Four 2D‐conjugated guest acceptors (X‐QTP‐4F) are developed by attaching different halogens into the 2D‐structured dibenzo[*f*,*h*]quinoxaline core. Among X‐QTP‐4F, Cl‐QTP‐4F shows a higher absorption coefficient, optimized molecular packing, suitable cascade energy levels, and complementary absorption with PM6:L8‐BO host. Thus, ternary devices achieved 19% efficiency, which is among the state‐of‐the‐art devices with 2D‐structured acceptors.

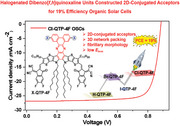

## 1 Introduction

Organic solar cells (OSCs) have received wide attention due to their prospective applications in flexible electronics, low‐cost power generators, and semitransparent facilities.^[^ ^1^, ^2^, ^3^, ^4^, ^5^ ^]^ Recently, thanks to some significant progresses in the molecular design of photovoltaic materials and the optimization of devices, OSCs have achieved power‐conversion‐efficiencies (PCEs) of exceeding 19%.^[^ ^6^, ^7^, ^8^, ^9^, ^10^, ^11^, ^12^, ^13^, ^14^, ^15^ ^]^ Looking back at the road‐map of OSCs recently, the innovative development of acceptor materials has greatly promoted photovoltaic performance.^[^ ^16^, ^17^, ^18^, ^19^, ^20^ ^]^ So far, the most successful is Y‐series small‐molecule acceptors (Y‐SMAs), as the latest generation acceptor materials.^[^ ^21^, ^22^, ^23^, ^24^, ^25^ ^]^ Compared to the other typed SMAs such as ITIC series,^[^ ^26^ ^]^ Y‐SMAs has an unique molecular structure of A‐DA'D‐A (A is acceptor and D is donor), which are generally composed of a DA'D‐typed π‐fused core with strong quinoidal character flanked by two end‐groups with strong electron‐withdrawing ability.^[^ ^27^, ^28^, ^29^, ^30^ ^]^ When varying the building blocks of DA'D core and end‐groups, Y‐SMAs are allowed to finely manipulate molecular photoelectric properties, such as crystallinity, HOMO (highest occupied molecular orbital) and LUMO (lowest unoccupied molecular orbital) energy levels, near‐infrared (NIR) absorption, and mixed intermolecular interactions in both the horizontal and perpendicular directions for forming a highly ordered 3D‐packing network.^[^ ^31^, ^32^, ^33^ ^]^ Moreover, such a banana shaped molecular configuration and ordered 3D‐packing of Y‐SMAs can guarantee efficient charge generation and exciton separation with a small driving force in their OSCs.^[^ ^34^ ^]^ It thus significantly enhance charge transport and suppress non‐radiative recombination of OSCs, achieving a much lower energy loss (*E*_loss_).^[^ ^35^, ^36^, ^37^ ^]^ On the other hands, recently, the state‐of‐the‐art PCEs of approaching 20% have been obtained by developing Y‐SMAs based ternary OSCs.^[^ ^38^, ^39^, ^40^ ^]^ However, although great efforts have been performed to modify molecular structure of Y‐SMAs, there are still few reports on how to accurately design guest Y‐SMAs to finely match the reported high‐performance binary hosts for constructing efficient ternary OSCs.^[^ ^41^ ^]^ Thus, exploring a feasible molecular design strategy, to develop new guest Y‐SMAs that can simultaneously broaden absorption, improve charge transfer, decrease *E*_loss_, and optimize morphology of efficient binary host, is the key to further boost PCEs of OSCs.

Recently, a variety of strategies were used to optimize the molecular structure of Y‐SMAs for gaining improved photoelectric properties, such as the modifications of DA'D core, end‐group, and alkyl side‐chain.^[^ ^42^, ^43^, ^44^, ^45^ ^]^ Typically, 2D‐conjugated extension of A’ unit in the vertical outward direction of DA'D core has been confirmed to be an effective approach to develop high‐performance Y‐SMAs,^[^ ^46^ ^]^ mainly due to the following 4 advantages. i) It can regulate the electron‐donating ability of the DA'D core to tailor intramolecular charge transfer (ICT) effect, thus enhancing NIR‐absorption and adjusting molecular energy levels;^[^ ^47^, ^48^ ^]^ ii) It allows to obtain a rigid and planar molecular geometry to improve intermolecular interaction, thus achieving order molecular stacking and optimized fibril network morphology;^[^ ^49^, ^50^ ^]^ iii) It can modulate molecular crystallization behavior and packing mode for offering optimal electron transport channel;^[^ ^51^, ^52^ ^]^ iv) It also suppresses molecular energetic disorder and reduce Urbach energy, resulting in the decreased *E*_loss_ in OSCs.^[^ ^53^, ^54^ ^]^ To satisfy the above preponderances, the A’ unit in the DA'D core of Y‐SMAs such as Y6 often be converted from benzothiadiazole (BT) to 2D‐conjugation extended quinoxaline (Qx)‐fused derivatives.^[^ ^55^ ^]^ For example, Zhu et al. designed 2D‐structured Y‐SMAs (AQx‐18) using the above strategy.^[^ ^56^ ^]^ When blending it with D18 to fabricate binary OSCs, a PCE of 18.2% was obtained, which is superior to its parental L8‐BO. Moreover, He et al. developed two 2D‐conjugated Y‐SMAs (YB2B and YB2T) by incorporating dibromodibenzo(or thieno)[*f*,*h*]quinoxaline derivatives as the A’ units,^[^ ^57^ ^]^ offering an increased PCE from 10.9% to 17.1%. On the other hand, Ge et al. reported two isomeric 2D‐structured Y‐SMAs (QX‐α and QX‐γ) with dithienoquinoxaline derivatives as the A’ units.^[^ ^58^ ^]^ In their ternary OSCs, QX‐α achieved a higher PCE of >19.0% compared to that of QX‐γ (18.3%). So far, the Qx‐fused derivatives as the A’ units are versatile and promising for constructing 2D‐structured Y‐SMAs with diverse optoelectronic properties. Compared with the other popular A’ units such as BT, benzoselenadiazole, and benzotriazole,^[^ ^59^, ^60^, ^61^ ^]^ 2D‐structured Qx‐fused A’ units used to lift molecular energy levels, which generally yield ambipolar charge transport characteristics and increase optical bandgap to reduce photon harvest.^[^ ^62^ ^]^ As a result, the above Y‐SMAs with a Qx‐fused A’ unit commonly have inevitable selection limitations of polymer donors, unbalanced carrier mobilities, and low short‐circuit current density (*J*_SC_) in OSCs.

To overcome these shortcomings, halogenation, as a feasible and effective strategy to optimize molecular photoelectric properties and active layer morphology, can be employed to modify these Qx‐fused A’ units of 2D‐conjugated Y‐SMAs, for achieving improved PCEs in OSCs.^[^ ^63^ ^]^ This is mainly due to that halogens have strong electronegativity and empty valence orbitals to form lone pair electrons, thus improving the electron‐withdrawing ability of resulting units, inter/intramolecular interactions, crystallinity, and electron mobility.^[^ ^64^, ^65^ ^]^ However, most efforts of halogen substitutions have focused on end‐groups of Y‐SMAs, and the influence of halogenation in the Qx‐fused A’ unit of 2D‐conjugated Y‐SMAs on molecular photoelectric properties has been rarely reported. On the other hand, different halogenation in the Qx‐fused A’ unit and how to manipulate the photovoltaic performance of 2D‐conjugated Y‐SMAs have not been systematically studied yet.

Herein, we developed a series of 2D‐conjugated Y‐SMAs named X‐QTP‐4F by attaching different halogens into the A’ units of 2D‐structured dibenzo[*f*,*h*]quinoxaline derivatives, such as halogen‐free H‐QTP‐4F, chlorinated Cl‐QTP‐4F, brominated Br‐QTP‐4F, and iodinated I‐QTP‐4F. Then, the effects of halogenation at Qx‐fused dibenzo[*f*,*h*]quinoxaline units on the molecular optoelectronic properties and photovoltaic performances of X‐QTP‐4F were systematically investigated. Among them, Cl‐QTP‐4F has a higher absorption coefficient, optimized molecular crystallinity and packing, suitable cascade energy levels, and complementary absorption with PM6:L8‐BO host. Moreover, among ternary PM6:L8‐BO:X‐QTP‐4F blends, PM6:L8‐BO:Cl‐QTP‐4F obtains a more uniform and size‐suitable fibrillary network morphology, improved molecular crystallinity and packing, as well as optimized vertical phase distribution, thus boosting charge generation, transport, extraction, and suppressing energy loss (*E*_loss_) of OSCs. Consequently, the PM6:L8‐BO:Cl‐QTP‐4F‐based OSCs achieved an impressive PCE of 19.0%, which is among the state‐of‐the‐art OSCs based on 2D‐conjugated Y‐SMAs and superior to these devices based on PM6:L8‐BO host (17.70%) and with guests of H‐QTP‐4F (18.23%), Br‐QTP‐4F (18.39%), and I‐QTP‐4F (17.62%). Our work highlights that the halogenation in dibenzo[*f*,*h*]quinoxaline cores of 2D‐conjugated Y‐SMA guests is a promising strategy to gain efficient ternary OSCs.

## 2 Results and Discussion

**Scheme 1:**
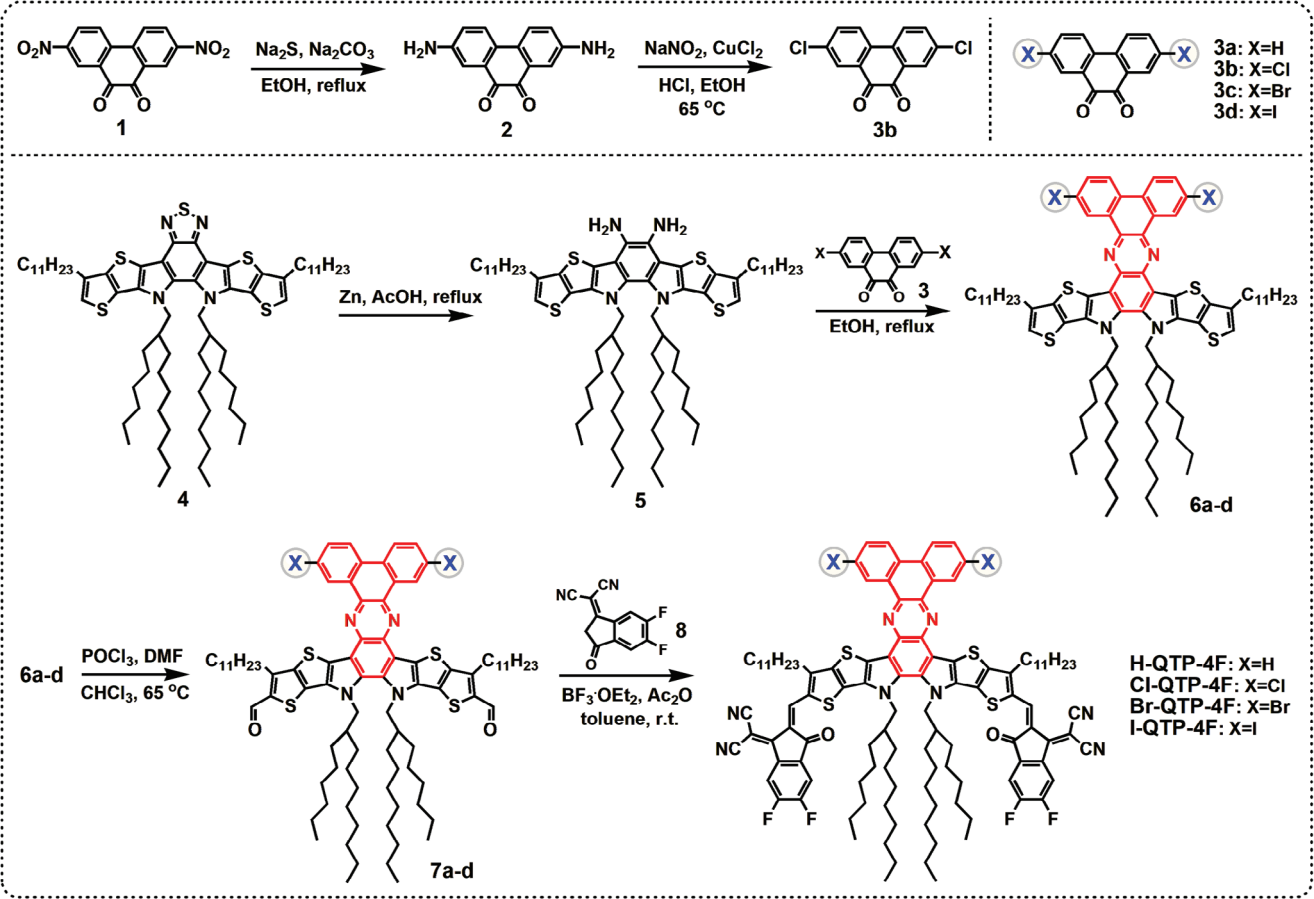
Synthetic routes and molecular structures of X‐QTP‐4F guest acceptors.

The synthetic routes and chemical structures of 2D‐conjugated X‐QTP‐4F guest acceptors without/with different halogenation in the A’ units of Qx‐fused dibenzo[*f*,*h*]quinoxaline derivatives were depicted in **Scheme** **1**. The commercially available precursor 4 was reacted with zinc powder and acetic acid to afford diamine compound 5. Without further purification, compound 5 was directly Cyclo‐condensated with 3a‐d to obtain 2D‐structured dibenzo[*f*,*h*]quinoxaline derivatives (named 6a‐d) with different halogenation. Compounds 7a‐d were then obtained from 6a‐d via a Vilsmeier‐Haack reaction. Finally, a Lewis acid‐catalyzed Knoevenagel condensation reaction was utilized to synthesize the target X‐QTP‐4F. The chemical structures of X‐QTP‐4F and related key intermediate products were verified by ^1^H/^13^C NMR and/or mass spectrometry (Figures S1–S18, Supporting Information).

**Figure 1:**
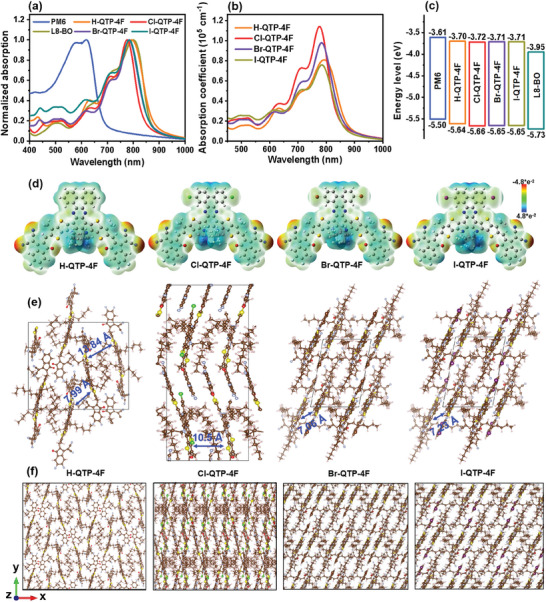
a) Normalized absorption spectra of active layer material films. b) Extinction coefficient of X‐QTP‐4F films. c) Diagrams of HOMO and LUMO levels of active layer materials. d) ESP distribution on the molecular models of X‐QTP‐4F. Packing patterns of X‐QTP‐4F from MD simulation: e) unit cells and f) supercells (Version 1), respectively.

UV–Vis absorption spectra of X‐QTP‐4F were measured to probe the effect of halogenation in the A’ units of Qx‐fused dibenzo[*f*,*h*]quinoxaline derivatives on molecular optical properties. As shown in Figure S19 (Supporting Information), in dilute chloroform, compared to halogen‐free H‐QTP‐4F, Cl‐QTP‐4F shows a blue‐shifted absorption mainly due to the weakened ICT effect between end‐groups and halogenated DA'D core. A similar phenomenon has been found in the previous works.^[^ ^66^ ^]^ Differently, Br‐QTP‐4F and I‐QTP‐4F exhibit significantly red‐shifted absorption compared to H‐QTP‐4F, which is probably because the heavy atom effect of bromine and iodine reduces the molecular solubility thus leading to the stronger self‐aggregation in solution state (Figure S20, Supporting Information). As displayed in **Figure** **1**a, halogenated X‐QTP‐4F series in neat films exhibit blue‐shifted absorption compared to H‐QTP‐4F one, which is also mainly due to the weakened ICT effect between end‐groups and halogenated DA'D core. On the other hand, as displayed in Figure 1b, Cl‐QTP‐4F film depicts a much higher extinction coefficient of 1.14 × 10^5^ cm^−1^ compared to the films of H‐QTP‐4F (0.81 × 10^5^ cm^−1^), Br‐QTP‐4F (0.98 × 10^5^ cm^−1^), and I‐QTP‐4F (0.76 × 10^5^ cm^−1^), implying its enhanced photon harvesting capacity. Benefiting from its higher absorption coefficient and more complementary absorption with PM6:L8‐BO host, Cl‐QTP‐4F among X‐TQP‐4F is the best guest candidate to construct efficient ternary OSCs.

The HOMO and LUMO energy levels of X‐QTP‐4F were measured by electrochemical cyclic voltammetry (Figure S21, Supporting Information), as −5.64/−3.70 eV for H‐QTP‐4F, −5.66/−3.72 eV for Cl‐QTP‐4F, −5.65/−3.71 eV for Br‐QTP‐4F, and −5.65/−3.71 eV for I‐QTP‐4F (Figure 1c), respectively. Notably, the similar LUMO levels of X‐QTP‐4F are higher than that of one from parental L8‐BO, which can form a cascade energy level alignment with PM6:L8‐BO system, thus offering more charge transport channels and efficient charge transfer at the D/A interface to reduce *E*_loss_ of OSCs.^[^ ^67^ ^]^ The HOMO and LUMO energy levels of PM6 were taken from the reported work as −5.50/−3.61 eV.^[^ ^27^ ^]^

The effects of halogenation in the A’ units of dibenzo[*f*,*h*]quinoxaline derivatives on the molecular geometry and frontier molecular orbitals of X‐QTP‐4F were probed by the density functional theory (DFT) calculation. As depicted in Figure S22 (Supporting Information), the optimal molecular geometries of X‐QTP‐4F guests are similar and nearly planar between the DA'D core and end groups. Wherein the electron clouds of HOMO orbitals are concentrated on the DA'D cores, and the electron clouds of LUMO orbitals are distributed on the molecular whole backbones. The halogenation in the 2D‐structured A’ units can finely adjust the molecular frontier orbital energy levels of X‐QTP‐4F series. The calculated HOMO/LUMO levels are −5.50/−3.48 eV for H‐QTP‐4F, −5.57/−3.52 eV for Cl‐QTP‐4F, −5.57/−3.52 eV for Br‐QTP‐4F, and −5.60/−3.54 eV for I‐QTP‐4F, respectively. Among X‐QTP‐4F, halogenated ones exhibit slightly down‐shifted HOMO/LUMO levels and increased bandgap. We also calculated the molecular electrostatic potential distribution (ESP) to investigate the structure‐property relationship. As illustrated in Figure 1d and X‐QTP‐4F series exhibit similar ESP distributions, where the main skeleton is more positive and the end groups are more negative. In the A’ unit of X‐QTP‐4F, the halogenated moiety is slightly more negative compared to the halogen‐free one, tending to have a weakened ICT effect and thus achieves a blue‐shifted absorption.

To further probe the effects of halogenation in the Qx‐fused A’ units on intermolecular interaction and packing pattern of X‐QTP‐4F, a molecular dynamics (MD) simulation was carried out.^[^ ^68^, ^69^ ^]^ As shown in Figure 1e, in terms of the symmetry of X‐QTP‐4F in the unit cells, their space groups can be delineated as orthorhombic *D*_2_ for H‐QTP‐4F, orthorhombic *D*_2h_ for Cl‐QTP‐4F, monoclinic *C*_2_ for both Br‐QTP‐4F and I‐QTP‐4F, respectively. As we know *D*_2h_ has the highest symmetry, which includes eight symmetric operations, such as identity operation (*E*), three double rotation operations (3*C*_2_), spatial inversion operation (*i*), and three mirror operations (*σ*_h_ and 2*σ*_v_). H‐QTP‐4F shows a significantly reduced symmetry, which decreases to only four symmetric operations (*E* and 3*C*_2_), indicating that the mirror symmetry is completely broken compared to Cl‐QTP‐4F. Moreover, Br‐QTP‐4F and I‐QTP‐4F display further reduced symmetry, which only keeps *E* and *C*_2_. Normally, Cl‐QTP‐4F with the highest symmetry tends to have stronger intermolecular order interaction. On the other hand, compared to H‐QTP‐4F, Br‐QTP‐4F, and I‐QTP‐4F with the unit cells embodying four molecules, Cl‐QTP‐4F has a unique unit cell with twelve molecules. More molecules in one unit cell can provide more opportunities for intermolecular interactions and thus more channels for charge transporting. As depicted in Figure 1f and Figures S23 and S24 (Supporting Information), the supercells of X‐QTP‐4F exhibit different packing patterns. Among them, Cl‐QTP‐4F has more orderly intermolecular packing and a unique 3D packing network of “head‐to‐tail” from end‐groups, “head‐to‐core” between end‐group and central core, and “core‐to‐core” from A’ units, resulting in the best charge‐transporting property.

**Figure 2:**
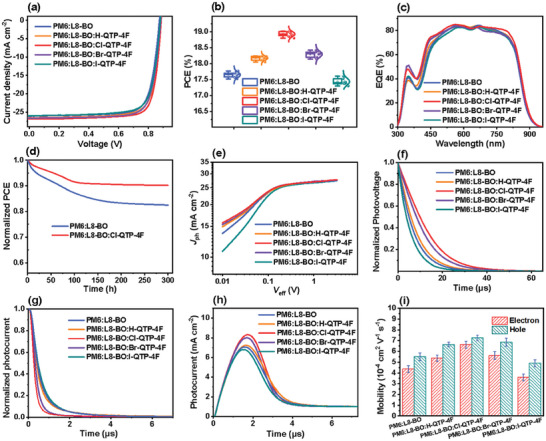
a) *J‐V* curves of the OSCs. b) The PCE statistics from the 10 independent OSCs. c) EQE spectra of the OSCs. d) Thermal stability of the OSCs under 50 °C in N_2_‐filled glove box. e) *J*_ph_‐*V*_eff_ curves, f) TPV decay kinetics, g) TPC decay kinetics, h) photo‐CELIV curves, and i) *µ*_e_ and *µ*_h_ values of the OSCs.

To study the effect of halogenation in the Qx‐fused dibenzo[*f*,*h*]quinoxaline derivatives as A’ units on the photovoltaic performance of 2D‐constructed X‐QTP‐4F guests in ternary devices based on PM6:L8‐BO host, the OSCs with a structure of ITO/PEDOT:PSS/active layer/PDIN/Ag were fabricated. The current density and voltage (*J‐V*) characteristics of the optimal OSCs under a simulated AM 1.5G illumination at 100 mW cm^−2^ were depicted in **Figure** **2**a, and the related photovoltaic parameters were summarized in Figure 2b and **Table** **1**. The OSCs based on binary PM6:L8‐BO yielded a PCE of 17.70% with an open‐circuit voltage (*V*_OC_) of 0.875 V, *J*_SC_ of 26.12 mA cm^−2^, and fill factor (FF) of 77.45%, which are comparable to the previous reports.^[^ ^70^, ^71^ ^]^ Adding Cl‐QTP‐4F guest into PM6:L8‐BO host, ternary OSCs achieved a much higher PCE of 19.0% due to the simultaneously boosted *V*_OC_ (0.887 V), *J*_SC_ (26.86 mA cm^−2^), and FF (79.75%), compared to the PM6:L8‐BO‐based parental devices. The PCE of 19.0% is among the top values in the reported OSCs with 2D‐conjugated Y‐SMAs. Similarly, ternary OSCs based on H‐QTP‐4F gained a higher PCE of 18.23% with a *V*_OC_ of 0.886 V, *J*_SC_ of 26.56 mA cm^−2^, and FF of 77.48%; ternary OSCs based on Br‐QTP‐4F offered an increased PCE of 18.39% with a *V*_OC_ of 0.883 V, *J*_SC_ of 26.65 mA cm^−2^, and FF of 78.16%. In contrast, ternary OSCs based on I‐QTP‐4F provided a slightly decreased PCE of 17.62% with a higher *V*_OC_ (0.881 V) but lower *J*_SC_ (25.96 mA cm^−2^) and FF (77.03%), which is probably due to that attaching iodine with big atomic radius and weight into X‐QTP‐4F leads to low intrinsic solubility and thus subsequently forms an excessively aggregated morphology.

**Table 1:** Photovoltaic parameters of the OSCs under AM 1.5G illumination at 100 mW cm^−2^.

Active layer	*V*_OC_ [V]	*J*_SC_ [mA cm^−2^]^a)^	FF [%]	PCE [%]^b)^
PM6:L8‐BO	0.875	26.12 (24.91)	77.45	17.70 (17.58 ± 0.21)
PM6:L8‐BO:H‐QTP‐4F	0.886	26.56 (25.01)	77.48	18.23 (18.05 ± 0.22)
PM6:L8‐BO:Cl‐QTP‐4F	0.887	26.86 (25.34)	79.75	19.00 (18.86 ± 0.19)
PM6:L8‐BO:Br‐QTP‐4F	0.883	26.65 (25.12)	78.16	18.39 (18.20 ± 0.23)
PM6:L8‐BO:I‐QTP‐4F	0.881	25.96 (24.83)	77.03	17.62 (17.45 ± 0.24)

^a)^ The integrated *J*_SC_ values were calculated from the EQE spectra;

^b)^ Average PCEs and the deviations calculated from 10 independent devices.

As depicted in Figure 2c, the external quantum efficiency (EQE) measurements were performed to verify the *J*_SC_ values of OSCs, and the corresponding integrated *J*_SC_ values were listed in Table 1. All the OSCs obtained high EQE response from 400 to 850 nm, and the related integrated *J*_SC_ values were calculated as 24.83–25.34 mA cm^−2^. The errors between the integrated *J*_SC_ values from EQE spectra and the measured *J*_SC_ values from *J‐V* plots are within 5%.

To evaluate the practical application potential of the champion OSCs based on PM6:L8‐BO:Cl‐QTP‐4F, the device's thermal stability was investigated under 50 °C in an N_2_‐filled glove box for 300 h. As shown in Figure 2d, the PM6:L8‐BO‐based device obtained 79.5% of its initial PCE, while the PM6:L8‐BO:Cl‐QTP‐4F‐based one achieved much better thermal stability with 88.2% of its initial PCE. The relatively high glass‐transition temperature (*T*_g_) delivers excellent operation stability under thermal stress. Based on the UV–Vis deviation metric (DMT) results (Figure S25, Supporting Information), the *T*_g_ values of L8‐BO and Cl‐QTP‐4F specimens are 88 and 115 °C, respectively. The higher *T*_g_ of Cl‐QTP‐4F implies that introducing Cl‐QTP‐4F guest into PM6:L8‐BO host can enable a relatively diffusion‐limited demixing of the morphology for active layers, and thus benefits for realizing long‐term thermal‐stable OSCs.

The curves of photocurrent density and effective voltage (*J*_ph_‐*V*_eff_) were plotted to explore the exciton and charge kinetics of OSCs (Figure 2e). The exciton dissociation and charge collection probabilities (*η*_d_ and *η*_c_) of the OSCs with different active layers can be calculated by the ratios of *J*_ph_/*J*_sat_ (*J*_sat_ is saturation current density) under short‐circuit current conditions and maximal power output conditions, as 95.14% and 87.90% for PM6:L8‐BO, 96.30% and 88.34% for PM6:L8‐BO:H‐QTP‐4F, 96.79% and 89.10% for PM6:L8‐BO:Cl‐QTP‐4F, 96.60% and 88.65% for PM6:L8‐BO:Br‐QTP‐4F, 94.90% and 87.74% for PM6:L8‐BO:I‐QTP‐4F, respectively. The superior *η*_d_ and *η*_c_ values of the OSCs based on PM6:L8‐BO:Cl‐QTP‐4F are well consistent with the increased *J*_SC_ and FF values.

Transient photovoltage (TPV) measurement was carried out to measure the photocarrier lifetime of devices for studying the charge recombination properties. As shown in Figure 2f, the PM6:L8‐BO:Cl‐QTP‐4F‐based devices obtained a longer photocarrier lifetime of 13.05 µs, as compared to these devices based on PM6:L8‐BO (6.93 µs), PM6:L8‐BO:H‐QTP‐4F (7.99 µs), PM6:L8‐BO:Br‐QTP‐4F (11.06 µs), and PM6:L8‐BO:I‐QTP‐4F (5.51 µs), respectively, implying lower charge recombination. Moreover, transient photocurrent (TPC) test was also performed to probe the charge extraction process of devices. As shown in Figure 2g, the Cl‐QTP‐4F‐based ternary devices gained a smaller charge extraction time of 0.24 *µ*s compared to these devices based on PM6:L8‐BO (0.48 µs), PM6:L8‐BO:H‐QTP‐4F (0.42 µs), PM6:L8‐BO:Br‐QTP‐4F (0.27 µs), and PM6:L8‐BO:I‐QTP‐4F (0.52 µs), respectively, suggesting a faster charge extraction process.

Moreover, the carrier mobilities of OSCs were estimated by using the photo‐induced carrier extraction in linearly increasing voltage (photo‐CELIV). As depicted in Figure 2h, the carrier mobilities of the OSCs based on PM6:L8‐BO, PM6:L8‐BO:H‐QTP‐4F, PM6:L8‐BO:Cl‐QTP‐4F, PM6:L8‐BO:Br‐QTP‐4F, and PM6:L8‐BO:I‐QTP‐4F were calculated as 1.30 × 10^−4^, 1.36 × 10^−4^, 2.23 × 10^−4^, 1.91 × 10^−4^, and 1.28 × 10^−4^ cm^2^ V^−1^ s^−1^, respectively. The higher carrier mobility of the Cl‐QTP‐4F‐based devices is beneficial for achieving superior PCE. Further, the space‐charge‐limited‐current (SCLC) method was used to independently measure the hole mobility (*µ*_h_) and electron mobility (*µ*_e_) values of devices. As shown in Figure 2i and Figure S26 (Supporting Information), the *µ*_h_ and *µ*_e_ values of the devices based on PM6:L8‐BO, PM6:L8‐BO:H‐QTP‐4F, PM6:L8‐BO:Cl‐QTP‐4F, PM6:L8‐BO:Br‐QTP‐4F, and PM6:L8‐BO:I‐QTP‐4F were calculated as 5.51 × 10^−4^/4.36 × 10^−4^, 6.64 × 10^−4^/5.37 × 10^−4^, 7.25 × 10^−4^/6.61 × 10^−4^, 6.85 × 10^−4^/5.61 × 10^−4^ and 4.90 × 10^−4^/3.59 × 10^−4^ cm^2^ V^−1^ s^−1^, with the *µ*_h_/*µ*_e_ ratios of 1.26, 1.24, 1.10, 1.22, and 1.36, respectively. The higher and more balanced *µ*_h_ and *µ*_e_ values of the Cl‐QTP‐4F‐based ternary devices could be responsible for the higher *J*_SC_ and FF values.

**Figure 3:**
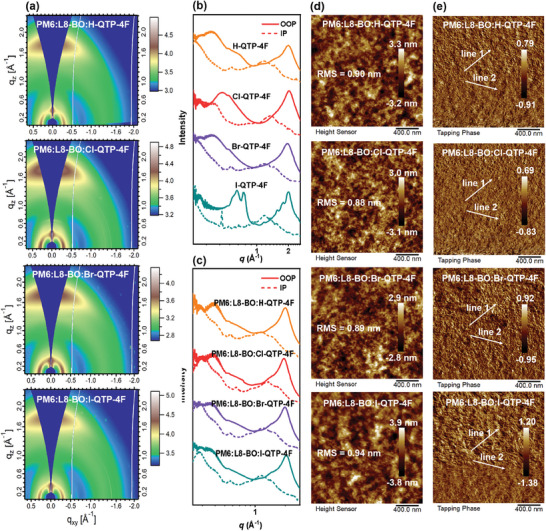
a) GIWAXS diffraction patterns of ternary blend films. Line‐cut profiles of GIWAXS images from b) X‐QTP‐4F neat films and c) related ternary blends. d) Height images and e) phase images of AFM measurements from ternary blends.

The grazing‐incidence wide‐angle X‐ray scattering (GIWAXS) measurement was utilized to investigate the molecular packing and orientation of the X‐QTP‐4F neat and blend films, as well as PM6:L8‐BO film (**Figure** **3**a–c and Figures S27 and S28, Supporting Information). The X‐QTP‐4F neat films show clear (010) peaks in the out‐of‐plane (OOP) direction and (100) peaks in the in‐plane (IP) direction, indicating the preferred “face‐on” orientation. The crystal coherence lengths (CCLs) of (010) peaks in the OOP direction for H‐QTP‐4F, Cl‐QTP‐4F, Br‐QTP‐4F, and I‐QTP‐4F are 17.3, 19.8, 18.2, and 21.7 Å, respectively, implying that introducing halogen into X‐QTP‐4F can improve molecular crystallinity of π‐π stacking. Three halogenated X‐QTP‐4F displays gradually increased π‐π stacking distances with the increase of halogen weight, which is mainly due to the increased halogen atomic radius. For the blend films, in the OOP direction, PM6:L8‐BO:Cl‐QTP‐4F film has an obviously sharper and stronger (010) diffraction peak with a similar π‐π stacking distance of 3.6 Å and much higher CCL of 19.0 Å, as compared to the PM6:L8‐BO (17.2 Å), PM6:L8‐BO:H‐QTP‐4F (17.6 Å), PM6:L8‐BO:Br‐QTP‐4F (18.9 Å), and PM6:L8‐BO:I‐QTP‐4F (17.1 Å) films. In the IP direction, PM6:L8‐BO:Cl‐QTP‐4F film displays a slightly stronger (100) diffraction peak with a similar lamellar stacking distance of 20.9 Å and a higher CCL of 62.1 Å, in comparison with the PM6:L8‐BO (55.9 Å), PM6:L8‐BO:H‐QTP‐4F (58.7 Å), PM6:L8‐BO:Br‐QTP‐4F (60.4 Å), and PM6:L8‐BO:I‐QTP‐4F (61.5 Å) films. The above results imply that introducing halogens can efficiently adjust molecular crystallinity and packing of X‐QTP‐4F, thus optimizing ternary active layers, especially PM6:L8‐BO:Cl‐QTP‐4F blend with better molecular crystallinity and packing.

To further study why Cl‐QTP‐4F guests can achieve better photovoltaic performances in ternary OSCs, atomic force microscopy (AFM) measurement was carried out to investigate the surface morphology of ternary active layers with different X‐QTP‐4F guests. For the AFM height images (Figure 3d; Figure S29a, Supporting Information), all the blends have smooth surface morphologies, and the corresponding root‐mean‐square (RMS) values are 0.93, 0.90, 0.88, 0.89, and 0.94 nm for PM6:L8‐BO, PM6:L8‐BO:H‐QTP‐4F, PM6:L8‐BO:Cl‐QTP‐4F, PM6:L8‐BO:Br‐QTP‐4F, and PM6:L8‐BO:I‐QTP‐4F, respectively. Among them, PM6:L8‐BO:Cl‐QTP‐4F depicts a smaller RMS value and cluster within the whole active layers, which is conducive to forming well‐distributed fibrillary interpenetrating network morphology. As depicted in Figure 3e and Figure S29b (Supporting Information) of the AFM phase images, PM6:L8‐BO:Cl‐QTP‐4F film exhibits a more uniform and size‐suitable tiny fiber‐like morphology. To make detailed comparison, the fibril widths of all blends were measured. The line profiles of the full‐width half maximum (FWHM) of the peaks are shown in Figures S29c and S30 (Supporting Information). The average FWHM values were calculated as 11.5, 10.8, 9.6, 10.2, and 12.4 nm for the PM6:L8‐BO, PM6:L8‐BO:H‐QTP‐4F, PM6:L8‐BO:Cl‐QTP‐4F, PM6:L8‐BO:Br‐QTP‐4F, and PM6:L8‐BO:I‐QTP‐4F blends, respectively. The PM6:L8‐BO:Cl‐QTP‐4F blend with a small surface roughness, and uniform and size‐suitable fibrillary network trends to achieve both high *J*_SC_ and FF values in OSCs.

**Figure 4:**
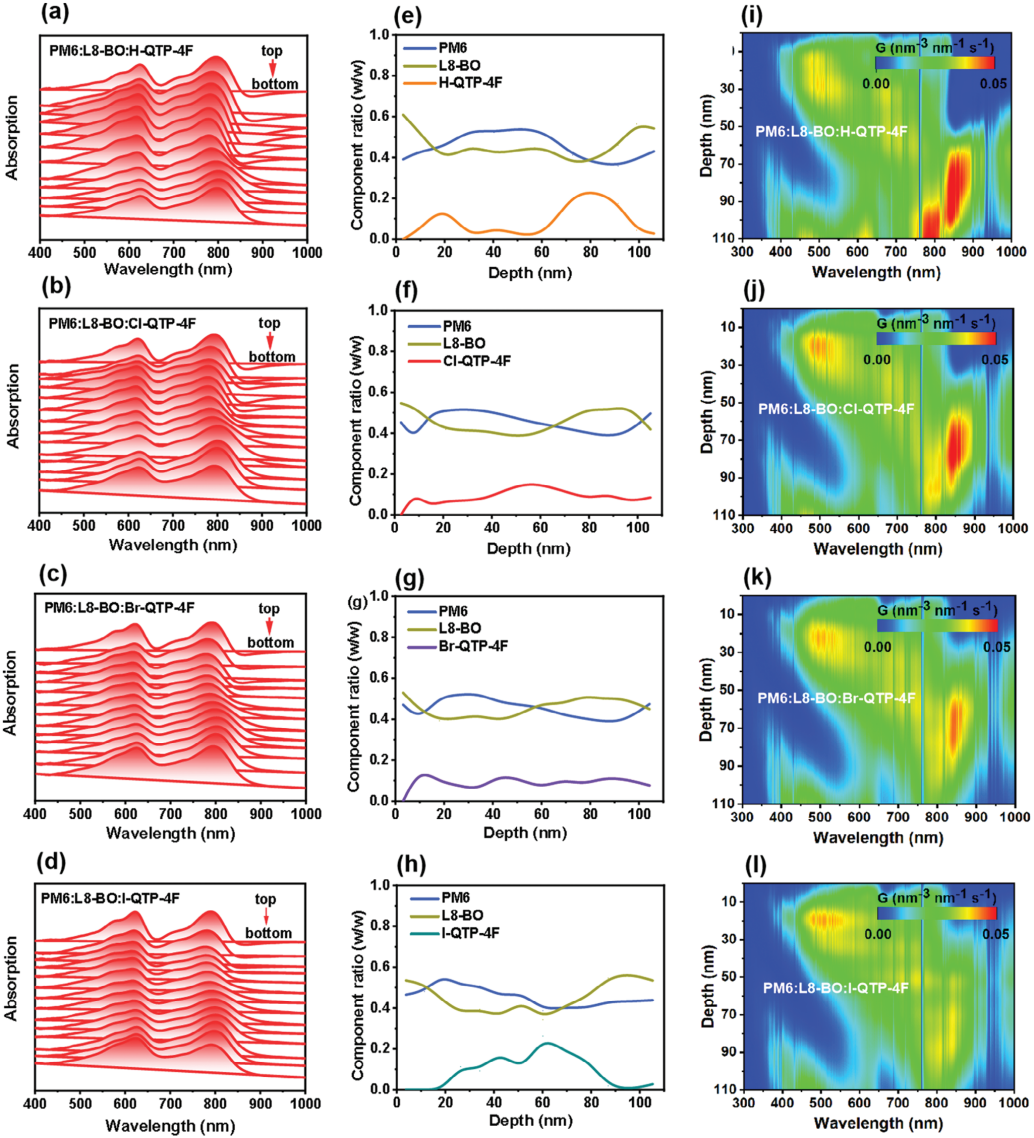
a–d) FLAS images of blends. For clarity, the spectra were re‐aligned along the absorption axis. e–h) Film‐depth‐dependent component distributions of PM6, L8‐BO, and X‐QTP‐4F in blends. i–l) Exciton generation contours as numerically simulated from the FLAS.

The effect of introducing X‐QTP‐4F guests into PM6:L8‐BO host on the vertical phase distributions of PM6 and L8‐BO was probed by performing film‐depth‐dependent light absorption spectrometry (FLAS). As shown in Figure S31a,b (Supporting Information), binary PM6:L8‐BO shows a compositional distribution of PM6 mainly at the top (film depth range of 20–45 nm) and L8‐BO mainly at the both top and bottom (film depth ranges of 0–20 and 50–100 nm). For the PM6:L8‐BO:X‐TQP‐4F blends (**Figure** **4**a–h), the compositional distribution of PM6 and L8‐BO show slight fluctuation. In PM6:L8‐BO:Cl‐QTP‐4F blend, due to the Cl‐QTP‐4F having well crystallinity, self‐assembly ability, favorable intermolecular interaction, and outstanding compatibility with L8‐BO, Cl‐QTP‐4F guest shows a more uniform distribution within whole active layer in the vertical direction compared to H‐QTP‐4F, Br‐QTP‐4F, and I‐QTP‐4F, which helps to a regular and well‐developed vertical phase separation with higher homogeneity. Such an optimized vertical distribution in PM6:L8‐BO:Cl‐QTP‐4F blend trends to improve exciton dissociation and suppress charge recombination of OSCs, thus achieving a boosted PCE.^[^ ^72^, ^73^ ^]^ Further, the exciton generation contours of active layers were numerically simulated using the transfer matrix method from the FLAS profiles (Figure 4i–l; Figure S31c, Supporting Information), and the related exciton generation rates (G) also were provided in Figures S31d and S32a–d (Supporting Information). In the contour figures, the exciton generation from the photoactive layers mostly occurs near the bottom part. Among these blends, PM6:L8‐BO:Cl‐QTP‐4F has superior maximum G values with wider and more balanced range distribution (film depth from 20 to 80 nm). Thus, the charge of ternary OSCs based on Cl‐QTP‐4F can more effectively generate and transport across the photoactive layer, which is also conducive to higher *J*_SC_ values.

**Figure 5:**
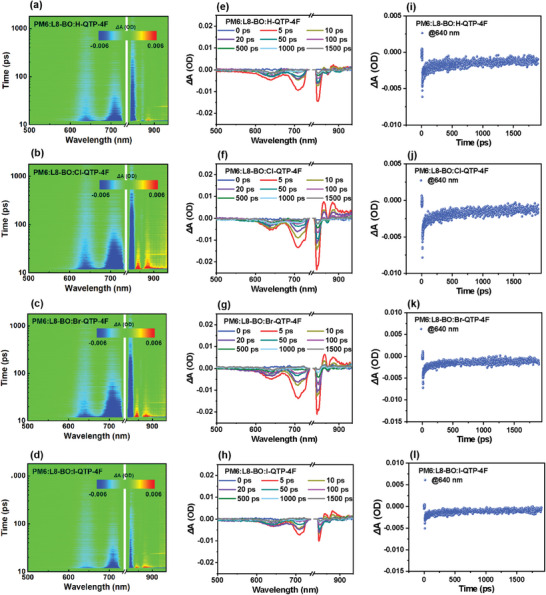
fs‐TA spectra presented in terms of Δ*T/T*. a–d) fs‐TA spectra profiles of ternary blends under a pump wavelength of 800 nm. e–h) fs‐TA spectra of ternary blends at different delay times. i–l) Hole transfer kinetic traces at 640 nm of ternary blends.

To probe the effect of introducing X‐QTP‐4F guests with different halogenation into PM6:L8‐BO host on hole transfer dynamics of OSCs, femtosecond transient absorption (fs‐TA) spectra of the PM6:L8‐BO (Figure S33, Supporting Information) and PM6:L8‐BO:X‐QTP‐4F blends were measured (**Figure** **5**a–h). A pump beam of 800 nm was chosen to solely excite the acceptors due to the significantly different absorption ranges between donor and acceptor components. Negative Δ*T*/*T* signals correspond to the ground state bleaching (GSB) of materials, whereas positive Δ*T*/*T* signals belong to the regions of excited state absorption (ESA).^[^ ^74^, ^75^ ^]^ As shown in Figure S33a,b (Supporting Information) and Figure 5a–h, a strong GSB peak at 710 nm and an ESA signal at 880 nm was observed immediately following excitation in all the blends, indicating the exciton formation of acceptors. Then, with the decay of the feature signals from acceptors, a new GSB peak appears at 640 nm, suggesting the hole transfer from acceptor to donor components. As depicted in Figure S33c (Supporting Information) and Figure 5i–l, the hole transfer kinetics of active layers were numerically described, via fitting the GSB signal at 640 nm of donor component, by the biexponential function with two lifetimes (*τ*_1_ and *τ*_2_). The τ_1_ is assigned to the ultrafast dissociation time at the D/A interfaces of the excitons formed in the acceptor components, and the τ_2_ is related to the time for excitons moving to the D/A interfaces.^[^ ^76^ ^]^ The τ_1_ and τ_2_ values were extracted as 3.6/29.4 ps for PM6:L8‐BO, 3.5/26.9 ps for PM6:L8‐BO:H‐QTP‐4F, 2.0/25.6 ps for PM6:L8‐BO:Cl‐QTP‐4F, 2.2/26.5 ps for PM6:L8‐BO:Br‐QTP‐4F, and 3.7/30.2 ps for PM6:L8‐BO:I‐QTP‐4F, respectively. Among these blends, PM6:L8‐BO:Cl‐QTP‐4F gained shorter τ_1_ and τ_2_ values, suggesting faster exciton dissociation and more efficient exciton diffusion, which is in accordance with its superior FF and PCE values in OSCs.

**Figure 6:**
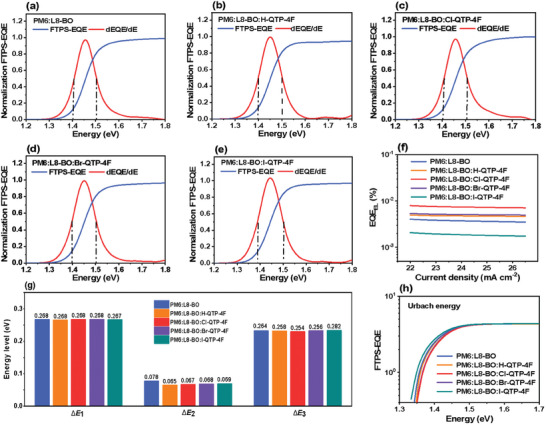
a–e) Normalized highly sensitive FTPS‐EQE curves and the derivatives of EQE spectra (dEQE/dE) of the OSCs. f) EQE_EL_ curves of the OSCs. g) Summary of schematic radiative and nonradiative energy loss. h) FTPS‐EQE curves of the OSCs.

As exhibited in **Figure** **6** and **Table** **2**, the detailed *E*_loss_ of OSCs has been studied to probe the effect of introducing X‐QTP‐4F guests into PM6:L8‐BO host on *V*_OC_. Generally, the *E*_loss_ of OSCs can be divided into three parts Δ*E*_1_, Δ*E*_2_, and Δ*E*_3_, as the following equation:^[^ ^77^ ^]^

(1) $$\begin{array}{ccc} E_{\mathrm{loss}} & = & E_{g}-V_{\mathrm{OC}}=\left(E_{g}-qV_{OC}^{SQ}\right)+\left(qV_{OC}^{SQ}-qV_{OC}^{rad}\right)\\ & & +\left(qV_{OC}^{rad}-qV_{\mathrm{OC}}\right)=\Delta E_{1}+\Delta E_{2}+\Delta E_{3} \end{array}$$

**Table 2:** Total *E*_loss_ and different contributions to *E*_loss_ of the OSCs.

Active layer	*E*_g_ [eV]	*E*_loss_ [eV]	$qV_{OC}^{SQ}$ [eV]	$qV_{OC}^{rad}$ [eV]	Δ*E*_1_ [eV]	Δ*E*_2_ [eV]	Δ*E*_3_ [eV]^a)^	Δ*E*_3_ [eV]^b)^
PM6:L8‐BO	1.454	0.610	1.186	1.108	0.268	0.078	0.233	0.264
PM6:L8‐BO:H‐QTP‐4F	1.451	0.591	1.183	1.118	0.268	0.065	0.232	0.258
PM6:L8‐BO:Cl‐QTP‐4F	1.452	0.589	1.184	1.117	0.268	0.067	0.230	0.254
PM6:L8‐BO:Br‐QTP‐4F	1.452	0.592	1.184	1.116	0.268	0.068	0.233	0.256
PM6:L8‐BO:I‐QTP‐4F	1.451	0.618	1.184	1.115	0.267	0.069	0.234	0.282

^a)^ Δ*E*_3_ calculated from $V_{OC}^{rad}$ − *qV*_OC_;

^b)^ Δ*E*_3_ calculated from the EQE_EL_ measured using a silicon detector.

Δ*E*_1_ is the radiative recombination loss above the bandgap, which can be defined as *E*_g_ − *q* $V_{OC}^{SQ}$, where *E*_g_ is the bandgap of OSCs estimated from the intersection between the highly sensitive Fourier transform photocurrent spectroscopy EQE (FTPS‐EQE) curve and the EL curve (Figure 6a–e), and $V_{OC}^{SQ}$ is the maximum theoretical *V*_OC_ using the Shockley‐Queisser model. In this work, all the OSCs obtained comparable Δ*E*_1_ values of 0.267–0.268 eV. Δ*E*_2_ represents the radiative recombination loss below the bandgap, which is equal to *q* $V_{OC}^{SQ}-qV_{OC}^{rad}$, where $V_{OC}^{rad}$ is the *V*_OC_ when there is only radiative recombination. Herein, the ternary OSCs with X‐QTP‐4F guests gained lower Δ*E*_2_ values of 0.065‐0.069 eV compared to the parental binary OSCs (0.078 eV).

Δ*E*_3_ is the non‐radiative recombination loss, which can be calculated by a formula of *q* $V_{OC}^{rad}$ − *qV*_OC_. As summarized in Table 2, all the OSCs offered similar Δ*E*_3_ values of 0.230–0.234 eV, while the PM6:L8‐BO:Cl‐QTP‐4F‐based device achieved a slightly smaller one. On the other hand, Δ*E*_3_ is connected to the electroluminescence quantum efficiency (EQE_EL_) of OSCs, which can be also estimated by the equation of −*k*Tln(EQE_EL_). As displayed in Figure 6f, the PM6:L8‐BO:Cl‐QTP‐4F‐based OSCs have a higher EQE_EL_ compared to the PM6:L8‐BO‐based devices with H‐QTP‐4F, Br‐QTP‐4F, or I‐QTP‐4F guests, leading to a smaller Δ*E*_3_ of 0.254 eV, which matches well with the Δ*E*_3_ variation tendency calculated from *q* $V_{OC}^{rad}$ − *qV*_OC_. Therefore, among the above OSCs, the Cl‐QTP‐4F‐based ternary OSCs obtained a smaller total *E*_loss_ of 0.565 eV. Furthermore, the Urbach energy of devices was analyzed to gain insight into the energetic disorder via the exponential fitting of FTPS‐EQE spectra. As depicted in Figure 6h, the Cl‐QTP‐4F‐based ternary OSCs offered smaller Urbach energy of 23.1 meV compared to the devices based on PM6:L8‐BO (24.4 meV), PM6:L8‐BO:H‐QTP‐4F (23.6 meV), PM6:L8‐BO:Br‐QTP‐4F (23.5 meV), and PM6:L8‐BO:I‐QTP‐4F (24.9 meV). The above results indicate that introducing Cl‐QTP‐4F guest into PM6:L8‐BO host can efficiently suppress the *E*_loss_ and energetic disorder of OSCs, achieving a higher *V*_OC_.

## 3 Conclusion

Four 2D‐conjugated Y‐SMAs named X‐QTP‐4F were developed by attaching different halogens into the 2D‐structured A’ units of dibenzo[*f*,*h*]quinoxaline derivatives. Among them, Cl‐QTP‐4F has a higher absorption coefficient, optimized molecular crystallinity and packing, suitable cascade energy levels, and complementary absorption with PM6:L8‐BO host. Among ternary PM6:L8‐BO:X‐QTP‐4F blends, PM6:L8‐BO:Cl‐QTP‐4F shows a more uniform and size‐suitable fibrillary network morphology, improved molecular crystallinity and packing, and optimized vertical phase distribution, thus boosting charge transfer, transport, extraction, and suppressing *E*_loss_ of OSCs. Consequently, the Cl‐QTP‐4F‐based ternary OSCs achieved an impressive PCE of 19.0%, which is among the top values in the reported OSCs based on 2D‐conjugated Y‐SMAs and superior to these OSCs based on PM6:L8‐BO host (17.70%) and with guest acceptors of H‐QTP‐4F (18.23%), Br‐QTP‐4F (18.39%), and I‐QTP‐4F (17.62%). Our work indicates that halogenation in the 2D‐structured dibenzo[*f*,*h*]quinoxaline core of Y‐SMA guests is a promising strategy for gaining efficient ternary OSCs.

## Conflict of Interest

The authors declare no conflict of interest.

## Supporting information

Supporting Information

Supplemental Table 1

## Data Availability

The data that support the findings of this study are available from the corresponding author upon reasonable request.
