# Supplementary material for: Halogenated Dibenzo[f,h]quinoxaline Units Constructed 2D‐Conjugated Guest Acceptors for 19% Efficiency Organic Solar Cells
Source: Adv Sci (Weinh). 2024 Jun 17;11(31):2403334. doi: 10.1002/advs.202403334 (PMC11336942; doi:10.1002/advs.202403334)
Supplement: Supplementary file 1 — Supporting Information [file ADVS-11-2403334-s002.docx]

Supporting Information

Halogenated Dibenzo[f,h]quinoxaline Units Constructed 2D-Conjugated Guest Acceptors for 19% Efficiency Organic Solar Cells

Jingshun Gao,^1,2^ Hairui Bai,^1,*^ Ping Li,^1^ Yibo Zhou,^1,3^ Wenyan Su,^3^ Chang Liu,^1^ Xiaoxiao Li,^4^ Yue Wu,^4^ Bin Hu,^5^ Zezhou Liang,^6^ Zhaozhao Bi,^1^ Xiong Li,^7^ Lihe Yan,^6^ Huiling Du,^3^ Guanghao Lu,^5^ Chao Gao,^8^ Kun Wang,^2,*^ Yuhang Liu,^1^ Wei Ma,^1,*^ and Qunping Fan^1,*^

^1^ J. Gao, H. Bai, P. Li, Y. Zhou, C. Liu, Z. Bi, Y. Liu, W. Ma, Q. Fan

State Key Laboratory for Mechanical Behavior of Materials, Xi’an Jiaotong University, Xi’an 710049, China. E-mail: baihairui@xjtu.edu.cn; msewma@xjtu.edu.cn; qunping@xjtu.edu.cn

^2^ J. Gao, K. Wang

School of Materials and Chemical Engineering, Zhongyuan University of Technology, Zhengzhou 451191, China. E-mail: kwang@zut.edu.cn

^3^ Y. Zhou, W. Su, H. Du

School of Materials Science and Engineering, Xi’an University of Science and Technology, Xi’an 710054, China

^4^ X. Li, Y. Wu

Laboratory of Advanced Optoelectronic Materials, Suzhou Key Laboratory of Novel Semiconductor-Optoelectronics Materials and Devices, College of Chemistry Chemical Engineering and Materials Science, Soochow University, Jiangsu, Suzhou 215123, China

^5^ B. Hu, G. Lu

Frontier Institute of Science and Technology, Xi’an Jiaotong University, Xi’an 710054, China

^6^ Z. Liang, L. Yan

Key Laboratory for Physical Electronics and Devices of the Ministry of Education & Shaanxi, Key Lab of Photonic Technique for Information, School of Electronics Science & Engineering, Faculty of Electronic and Information Engineering, Xi’an Jiaotong University, Xi’an 710049, China

^7^ X. Li

Department of Physics, Beijing Technology and Business University, Beijing 100048, China.

^8^ C. Gao

Xi'an Key Laboratory of Liquid Crystal and Organic Photovoltaic Materials, State Key Laboratory of Fluorine & Nitrogen Chemicals, Xi'an Modern Chemistry Research Institute, Xi'an 710065, China

J. Gao, H. Bai, and P. Li contributed equally to this work.

**Experimental Section**

**Materials and characterization**

All air and water-sensitive reactions were carried out under nitrogen. ^1^H NMR and ^13^C NMR spectra were collected with a Bruker 400 MHz AVANCE III spectrometer in CDCl_3_. The UV-vis absorption spectrum was measured by a Shimadzu UV-1780 Spectrophotometer. Cyclic voltammetry (CV) was probed on a Donghua DH7000C electrochemical workstation in an anhydrous acetonitrile solution of tetra-*n*-butylammonium hexafluoro-phosphate (Bu_4_NPF_6_) (0.1 M) with a scan rate of 50 mV s^-1^. A conventional three-electrode cell was used with a platinum plate working electrode, a platinum wire counter-electrode, and an Ag/AgCl reference electrode. Ferrocene/Ferrocenium (Fc/Fc^+^) used as the internal standard and their energy levels are assumed at -4.8 eV relative to vacuum. Atomic force microscopy (AFM) images were taken in tapping mode with a Bruker Innova scanning probe microscope. GIWAXS measurements were performed at beamline 7.3.3 at the Advanced Light Source. Samples were prepared on Si substrates using identical blend solutions as those used in devices. The 10 keV X-ray beam was incident at a grazing angle of 0.10-0.16°, selected to maximize the scattering intensity from the samples. The scattered x-rays were detected using a Dectris Pilatus 2 M photon counting detector. The crystal coherence length (CCL) was defined as CCL = 0.9×(2π/FWHM) (Å), where FWHM is the full width at half maximum of the corresponding diffraction peak.^1^

***Computational Details***: Theoretical simulation DFT were carried out at the B3LYP/6-31G(d,p) level using Gaussian. To gain a deeper understanding of the effect of alkyl-chain engineering on intermolecular interactions and packing patterns, the crystal structures of the studied acceptors were predicted by carrying out molecular dynamics (MD) simulations. Firstly, the single molecule was optimized using the Dmol3 program with a fine accuracy setting. Additionally, electrostatic potential and population analysis were performed. Subsequently, crystal structure prediction was carried out using the Perdew-Burke-Ernzerhof (PBE) exchange-correlation energy functional and the Dreiding force field. This process was conducted in the polymorph module, which has proven to be a valuable method for predicting crystal structures. All calculations were performed using Materials Studio (One Molecular Simulation Software, <https://www.accelrys.com).>

***Materials*:** Polymer donor PM6 with a *M*_n_ of 36.0 kDa (Lot# YN425C) and PDIN^[2]^ were purchased from Solarmer Materials Inc. Monomer 3a, 3c, 3d, were purchased from Zhengzhou alfachem. L8-BO were synthesized according to the previous reports.^3^ Monomer 3b, H-QTP-4F, Cl-QTP-4F, Br-QTP-4F and I-QTP-4F were developed according to the following procedures:

***Monomer 3b*:** In a 50 mL round-bottom flask, **1** in methanol (10 mL) was added to the hot water (10 ml) solution of Na_2_CO_3_ (0.36 g, 3.4 mmol) and Na_2_S (0.35 g, 4.48 mmol). Then, the resulting reaction mixturere stirred at 90 ℃ for 1 h. After reaction, the crude product **2** was collected by filtration and then dried as a dark blue solid (0.71 g, 87 %). No further with any purification, **2** (0.35 g, 1.46 mmol) was added to ethanol (10 mL), and then slowly added to a solution of sodium nitrite (NaNO_3_, 0.23 g, 3.3 mmol) and copper chloride (CuCl_2_, 0.58 g, 4.35 mmol) in hydrochloric acid at 0 °C. Then, the resulting reaction mixturere stirred at 65 ℃ for 2 hours. After being cooled to room temperature, the mixture was quenched with water and then extracted with dichloromethane (DCM). The organic layers were combined and washed with saturated brine solution and dried over anhydrous sodium sulfate (Na_2_SO_4_). After removal of the solvent, the crude product was purified by silica gel column chromatography, eluting with petroleum ether (PE)/DCM (1:1) to obtai product **3b** as an orange solid. (0.28 g, 68%).^1^H NMR (400 MHz, DMSO-*d*_6_) *δ* 8.69 (s, 2H), 8.67 (s, 2H), 8.54 (d, *J* = 8.7 Hz, 2H).

***6a***: In a 50 mL round-bottom flask, **4** (0.832 g, 0.716 mmol) in acetic acid (16 mL) was added zinc powder (0.930 g, 14.32 mmol). The reaction was then refluxed 4 h at 90 ℃ under N_2_ atmosphere. After being cooled to room temperature, the mixture was quenched with saturated aqueous solution of NaCl and then extracted with DCM. The organic layers were combined and washed with saturated brine solution and dried over anhydrous Na_2_SO_4_. The solvent was removed by rotary evaporation to give the crude product **5** (0.416 g, 0.358 mmol) as an faint yellow oil. No further with any purification, crude product **5** and phenanthrene-9,10-dione **3a** (0.076 g, 0.358 mmol) were added to ethanol (10 mL) and acetic acid (10 mL) solution. The reaction mixturere vigorously stirred at 100 °C for overnight under N_2_ atmosphere. After being cooled to room temperature, the mixture was quenched with water and then extracted with DCM. The organic layers were combined and washed with saturated brine solution and dried over anhydrous Na_2_SO_4_, and then filtered and collected solvent. The solvent was removed by rotary evaporation to give the crude product as a red oil, which was purified by column chromatography on silica gel with PE/DCM (v/v, 8:1) as eluent to afford **6a** as a red viscous liquid (0.136 g, 30%).^1^H NMR (400 MHz, CDCl3) *δ* 9.74 (d, *J* = 7.2 Hz, 2H), 8.69 (d, *J* = 8.1 Hz, 2H), 7.92 (t, *J* = 7.5 Hz, 2H), 7.83 (t, *J* = 7.0 Hz, 2H), 7.04 (s, 2H), 4.69 (d, *J* = 7.7 Hz, 4H), 2.93 (t, *J* = 7.6 Hz, 4H), 2.18 (s, 2H), 1.98 - 1.91 (m, 4H), 1.28-0.85 (m, 80H), 0.78-0.74 (m, 6H), 0.73 - 0.57 (m, 12H).

***6b***: The crude product **5** (0.416 g, 0.358 mmol) and 2,7-dichlorophenanthrene-9,10-dione **3b** (0.05 g, 0.179 mmol) were added to ethanol (10 mL) and acetic acid (10 mL) solution. The reaction mixturere vigorously stirred at 100 °C for overnight under N_2_ atmosphere. After being cooled to room temperature, the mixture was quenched with water and then extracted with DCM. The organic layers were combined and washed with saturated brine solution and dried over anhydrous Na_2_SO_4_, and then filtered and collected solvent. The solvent was removed by rotary evaporation to give the crude product as a red oil, which was purified by column chromatography on silica gel with PE/DCM (v/v, 8:1) as eluent to afford **6b** as a red viscous liquid (0.141 g, 28%). ^1^H NMR (400 MHz, CDCl_3_) *δ* 10.54 (d, *J* = 2.5 Hz, 2H), 8.72 (d, *J* = 9.1 Hz, 2H), 8.57-8.55 (m, 2H), 7.08 (s, 2H), 4.74 (d, *J* = 7.9 Hz, 4H), 2.96 (t, *J* = 7.6 Hz, 4H), 2.23 - 2.19 (m, 2H), 1.97 (d, *J* = 7.4 Hz, 4H), 1.26 - 0.99 (m, 80H), 0.72 (d, *J* = 7.1 Hz, 6H), 0.70 - 0.62 (m, 12H).

***6c***: The crude product **5** (0.416 g, 0.358 mmol) and 2,7-dibromophenanthrene-9,10-dione **3c** (0.065 g, 0.179 mmol) were added to ethanol (10 mL) and acetic acid (10 mL) solution. The reaction mixturere vigorously stirred at 100 °C for overnight under N_2_ atmosphere. After being cooled to room temperature, the mixture was quenched with water and then extracted with DCM. The organic layers were combined and washed with saturated brine solution and dried over anhydrous Na_2_SO_4_, and then filtered and collected solvent. The solvent was removed by rotary evaporation to give the crude product as a red oil, which was purified by column chromatography on silica gel with PE/DCM (v/v, 8:1) as eluent to afford **6c** as a red viscous liquid (0.117 g, 22%).^1^H NMR (400 MHz, CDCl3) *δ* 9.65 (s, 2H), 8.01 (d, *J* = 8.1 Hz 2H), 7.68 (d, *J* = 8.6 Hz, 2H), 7.07 (s, 2H), 4.75 (d, *J* = 7.8 Hz, 4H), 2.97 (t, *J* = 7.7 Hz, 4H), 2.24 (s, 2H), 1.99 (d, *J* = 7.5 Hz, 4H), 1.32 - 0.92 (m, 80H), 0.80 (d, *J* = 6.6 Hz, 6H), 0.75 - 0.59 (m, 12H).

***6d***: The crude product **5** (0.416 g, 0.358 mmol) and 2,7-diiodophenanthrene-9,10-dione **3d** (0.164 g, 0.358 mmol) were added to ethanol (10 mL) and acetic acid (10 mL) solution. The reaction mixturere vigorously stirred at 100 °C for overnight under N_2_ atmosphere. After being cooled to room temperature, the mixture was quenched with water and then extracted with DCM. The organic layers were combined and washed with saturated brine solution and dried over anhydrous Na_2_SO_4_, and then filtered and collected solvent. The solvent was removed by rotary evaporation to give the crude product as a red oil, which was purified by column chromatography on silica gel with PE/DCM (v/v, 8:1) as eluent to afford **6d** as a red viscous liquid (0.120 g,21%). ^1^H NMR (400 MHz, CDCl3) *δ* 9.79 (s, 2H), 7.76 (d, *J* = 9.2 Hz, 2H), 7.72 (d, *J* = 8.7 Hz, 2H), 7.08 (s, 2H), 4.76 (d, *J* = 7.7 Hz, 4H), 3.00 (t, *J* = 7.7 Hz, 4H), 2.26 (s, 2H), 2.06 - 2.00 (m, 4H), 1.30 - 0.88 (m, 80H), 0.79 (d, *J* = 14.5 Hz, 6H), 0.75 - 0.58 (m, 12H).

***7a***: In an ice bath, POCl_3_ (0.5 mL) was dropwise added into a solution of anhydrous DMF (0.5 mL) and kept 10 minutes under the protection of argon, and then kept additional 30 minutes at room temperature under stirring. After that, **6a** (0.10 g, 0.075 mmol) dissolved in chloroform (4 mL) was added into the reaction mixture and then heated at 65 ℃ for overnight, cooled to room temperature, quenched with saturated aqueous solution of sodium acetate, and extracted with DCM. The combined extracts were washed with brine, dried over anhydrous Na_2_SO_4_, and then filtered and collected solvent. The solvent was removed by rotary evaporation to yield the crude product, which was then purified by column chromatography on silica gel with PE/DCM (v/v, 2:1) as eluent to afford **7a** as a yellow viscous liquid (0.080 g, 76%). ^1^H NMR (400 MHz, CDCl_3_) *δ* 10.12 (d, *J* = 6.8 Hz, 2H), 9.63 (d, *J* = 6.9 Hz, 2H), 8.64 (d, *J* = 7.9 Hz, 2H), 7.86 (t, *J* = 7.5 Hz, 2H), 7.78 (t, *J* = 7.6 Hz, 2H), 4.81 (d, *J* = 7.8 Hz, 4H), 3.38 (t, *J* = 7.6 Hz, 4H), 2.31 - 2.14 (m, 2H), 2.11 - 2.04 (m, 4H), 1.39 - 1.00 (m, 80H), 0.79 - 0.76 (m, 6H), 0.75 - 0.66 (m, 12H).

***7b***: In an ice bath, POCl_3_ (0.5 mL) was dropwise added into a solution of anhydrous DMF (0.5 mL) and kept 10 minutes under the protection of argon, and then kept additional 30 minutes at room temperature under stirring. After that, 3b (0.105 g, 0.075 mmol) dissolved in chloroform (4 mL) was added into the reaction mixture and then heated at 65 ℃ for overnight, cooled to room temperature, quenched with saturated aqueous solution of sodium acetate, and extracted with DCM. The combined extracts were washed with brine, dried over anhydrous Na_2_SO_4_, and then filtered and collected solvent. The solvent was removed by rotary evaporation to yield the crude product, which was then purified by column chromatography on silica gel with PE/DCM (v/v, 2:1) as eluent to afford 4b as a red viscous liquid (0.105 g, 96%). ^1^H NMR (400 MHz, CDCl_3_) *δ* 10.58 (d, *J* = 2.5 Hz, 2H), 10.20 (s, 2H), 8.85 (d, *J* = 9.1 Hz, 2H), 8.69 - 8.66 (m, 2H), 4.75 (d, *J* = 7.8 Hz, 4H), 3.34 (t, *J* = 7.6 Hz, 4H), 2.19 (s, 2H), 2.09 - 2.03 (m, 4H), 1.26 - 0.93 (m, 80H), 0.79 - 0.71 (m, 6H), 0.70 - 0.60 (m, 12H).

***7c***: In an ice bath, POCl_3_ (0.5 mL) was dropwise added into a solution of anhydrous DMF (0.5 mL) and kept 10 minutes under the protection of argon, and then kept additional 30 minutes at room temperature under stirring. After that, 3c (0.112 g, 0.075 mmol) dissolved in chloroform (4 mL) was added into the reaction mixture and then heated at 65 ℃ for overnight, cooled to room temperature, quenched with saturated aqueous solution of sodium acetate, and extracted with DCM. The combined extracts were washed with brine, dried over anhydrous Na_2_SO_4_, and then filtered and collected solvent. The solvent was removed by rotary evaporation to yield the crude product, which was then purified by column chromatography on silica gel with PE/DCM (v/v, 2:1) as eluent to afford 4c as a yellow viscous liquid (0.135 g, 87%). ^1^H NMR (400 MHz, CDCl_3_) *δ* 10.20 (s, 2H), 9.68 (d, *J* = 2.1 Hz, 2H), 8.24 (d, *J* = 8.8 Hz, 2H), 7.78-7.82 (m, 2H), 4.76 (d, *J* = 7.9 Hz, 4H), 3.34 (t, *J* = 7.7 Hz, 4H), 2.19 (s, 2H), 2.11 - 2.03 (m, 4H), 1.27 - 0.86 (m, 80H), 0.80-0.75 (m, 6H), 0.74 - 0.60 (m, 12H).

***7d***: In an ice bath, POCl_3_ (0.5 mL) was dropwise added into a solution of anhydrous DMF (0.5 mL) and kept 10 minutes under the protection of argon, and then kept additional 30 minutes at room temperature under stirring. After that, 3d (0.120 g, 0.075 mmol) dissolved in chloroform (3 mL) was added into the reaction mixture and then heated at 65 ℃ for overnight, cooled to room temperature, quenched with saturated aqueous solution of sodium acetate, and extracted with DCM. The combined extracts were washed with brine, dried over anhydrous Na_2_SO_4_, and then filtered and collected solvent. The solvent was removed by rotary evaporation to yield the crude product, which was then purified by column chromatography on silica gel with PE/DCM (v/v, 2:1) as eluent to afford 7d as a yellow viscous liquid (0.108 g, 88%). ^1^H NMR (400 MHz, CDCl_3_) δ 10.13 (s, 2H), 9.89(s, 2H), 8.12 (d, *J* = 8.7 Hz, 2H), 7.96 (d, *J* = 8.6 Hz, 2H), 4.83 (d, *J* = 7.7 Hz, 4H), 3.40 (t, *J* = 7.9 Hz, 4H), 2.24 (d, *J* = 10.4 Hz, 2H), 2.15 - 2.10 (m, 4H), 1.38 - 1.28 (m, 80H), 0.83-0.80 (m, 6H), 0.79 - 0.68 (m, 12H).

***H-QTP-4F***: In a dry 25 mL flask, compounds of **4a** (60 mg, 0.045 mmol), **IC-2F** (31 mg, 0.135 mmol) were dissolved in toluene (4 mL). BF_3_∙OEt_2_ (0.1 mL, 0.81 mmol) and acetic anhydride (1.0 ml) were added, and the reaction mixture was stir at room temperature for 15 min. Then the mixture was poured into methanol (50 mL) followed by precipitation, and the sediments were collected. The resulting crude compound was purified by column chromatography on silica gel with PE/DCM (*v*/*v*=2:1) as eluent and then it was furtherly purified by crystallized two times using a mix solvent of chloroform/methanol (*v*/*v*=1:1) to give **H-QTP-4F** as a black solid (70 mg, 85%). ^1^H NMR (400 MHz, CDCl_3_) *δ* 9.55 (d, *J* = 7.8 Hz, 2H), 9.11 (s, 2H), 8.68 (d, *J* = 8.0 Hz, 2H), 8.42 - 8.41 (m, 2H), 7.93 - 7.87 (m, 4H), 7.68 - 7.55 (m, 2H), 4.86 (d, *J* = 7.5 Hz, 4H), 3.31 (t, *J* = 7.7 Hz, 4H), 2.28 (s, 2H), 1.97 - 1.89 (m, 4H), 1.28 - 0.96 (m, 80H), 0.83 - 0.80 (m, 6H), 0.71 - 0.65 (m, 12H). MALDI-TOF-MS (m/z): calcd for C_112_H_126_F_4_N_8_O_2_S_4_, 1819.88, found 1819.88.

***Cl-QTP-4F***: In a dry 25 mL flask, compounds of **4b** (66 mg, 0.045 mmol), **IC-2F** (31 mg, 0.135 mmol) were dissolved in toluene (4 mL). BF_3_∙OEt_2_ (0.1 mL, 0.81 mmol) and acetic anhydride (1.0 ml) were added, and the reaction mixture was stir at room temperature for 15 min. Then the mixture was poured into methanol (50 mL) followed by precipitation, and the sediments were collected. The resulting crude compound was purified by column chromatography on silica gel with PE/DCM (*v*/*v*=2:1) as eluent and then it was furtherly purified by crystallized two times using a mix solvent of chloroform/methanol (*v*/*v*=1:1) to give **Cl-QTP-4F** as a black solid (76 mg, 90%).^1^H NMR (600 MHz, CDCl_3_) *δ* 9.99 (s, 2H), 8.79 (s, 2H), 8.61 (t, *J* = 10.6 Hz, 4H), 7.87 (s, 2H), 7.66 (s, 2H), 5.20 (d, *J* = 118.2 Hz, 4H), 3.32 - 3.02 (s, 4H), 2.64 (s, 2H), 2.03 - 1.84 (m, 4H), 1.46 - 1.05 (m, 80H), 0.88 - 0.79 (m, 6H), 0.78-0.75 (m, 12H).MALDI-TOF-MS (m/z): calcd for C_112_H_124_Cl_2_F_4_N_8_O_2_S_4_, 1887.81, found 1887.82.

***Br-QTP-4F***: In a dry 25 mL flask, compounds of **4d** (70 mg, 0.045 mmol), **IC-2F** (31 mg, 0.135 mmol) were dissolved in toluene (4 mL). BF_3_∙OEt_2_ (0.1 mL, 0.81 mmol) and acetic anhydride (1.0 ml) were added, and the reaction mixture was stir at room temperature for 15 min. Then the mixture was poured into methanol (50 mL) followed by precipitation, and the sediments were collected. The resulting crude compound was purified by column chromatography on silica gel with PE/DCM (*v*/*v*=2:1) as eluent and then it was furtherly purified by crystallized two times using a mix solvent of chloroform/methanol (*v*/*v*=1:1) to give **Br-QTP-4F** as a black solid (58 mg, 65%). ^1^H NMR (400 MHz, CDCl_3_) *δ* 9.48 (s, 2H), 8.98 (s, 2H), 8.41 (d, *J* = 8.9 Hz, 2H), 8.22 (s, 2H), 7.94 - 7.91 (m, 2H), 7.60 (s, 2H), 4.91 (d, *J* = 6.6 Hz, 4H), 3.28 - 3.22 (t, 4H), 2.40 (s, 2H), 1.94 - 1.89 (m, 4H), 1.06 (m, 80H), 0.82 - 0.79 (m, 6H), 0.76-0.69 (m, 12H). MALDI-TOF-MS (m/z): calcd for C_112_H_124_Br_2_F_4_N_8_O_2_S_4_, 1974.70, found 1975.87.

***I-QTP-4F***: In a dry 25 mL flask, compounds of **4d** (75 mg, 0.045 mmol), **IC-2F** (31 mg, 0.135 mmol) were dissolved in toluene (4 mL). BF_3_∙OEt_2_ (0.1 mL, 0.81 mmol) and acetic anhydride (1.0 ml) were added, and the reaction mixture was stir at room temperature for 15 min. Then the mixture was poured into methanol (50 mL) followed by precipitation, and the sediments were collected. The resulting crude compound was purified by column chromatography on silica gel with PE/DCM (*v*/*v*=2:1) as eluent and then it was furtherly purified by crystallized two times using a mix solvent of chloroform/methanol (*v*/*v*=1:1) to give ***I-QTP-4F*** as a black solid (67 mg, 72%).^1^H NMR (400 MHz, CDCl_3_) *δ* 9.78 (s, 2H), 9.02 (s, 2H), 8.27 (d, *J* = 8.9 Hz, 4H), 8.12 - 8.09 (m, 2H), 7.61 (s, 2H), 4.89 (d, *J* = 7.1 Hz, 4H), 3.31 - 3.24 (t, 4H), 2.38 (s, 2H), 1.97 - 1.90 (m, 4H), 1.29 - 1.01 (m, 80H), 0.82 - 0.79 (m, 6H), 0.73 - 0.69 (m, 12H). MALDI-TOF-MS (m/z): calcd for C_112_H_124_F_4_I_2_N_8_O_2_S_4_, 2071.67, found 2071.18.

**Figure S1.** ^1^H NMR image of compound **1**.

**Figure S2.** ^1^H NMR image of compound **3b**.

**Figure S3.** ^1^H NMR image of compound **6a**.

**Figure S4.** ^1^H NMR image of compound **6b**.

**Figure S5.** ^1^H NMR image of compound **6c**.

**Figure S6.** ^1^H NMR image of compound **6d**.

**Figure S7.** ^1^H NMR image of compound **7a**.

**Figure S8.** ^1^H NMR image of compound **7b**.

**Figure S9.** ^1^H NMR image of compound **7c**.

**Figure S10.** ^1^H NMR image of compound **7d**.

**Figure S11.** ^1^H NMR image of **H-QTP-4F**.


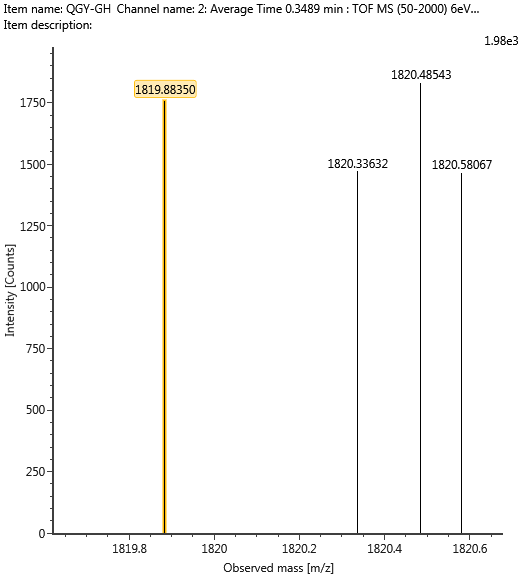


**Figure S12**. MS image of **H-QTP-4F**.

**Figure S13.** ^1^H NMR image of **Cl-QTP-4F**.


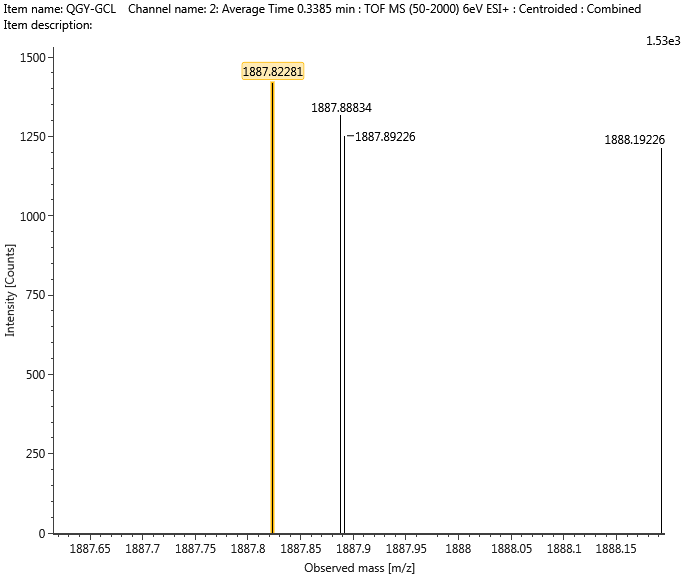


**Figure S14**. MS image of **Cl-QTP-4F**.

**Figure S15.** ^1^H NMR image of **Br-QTP-4F**.


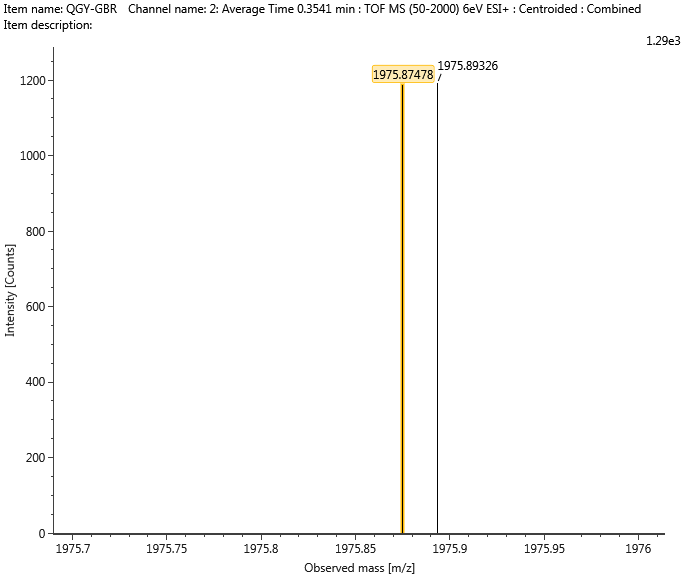


**Figure S16**. MS image of **Br-QTP-4F**.

**Figure S17.** ^1^H NMR image of **I-QTP-4F**.


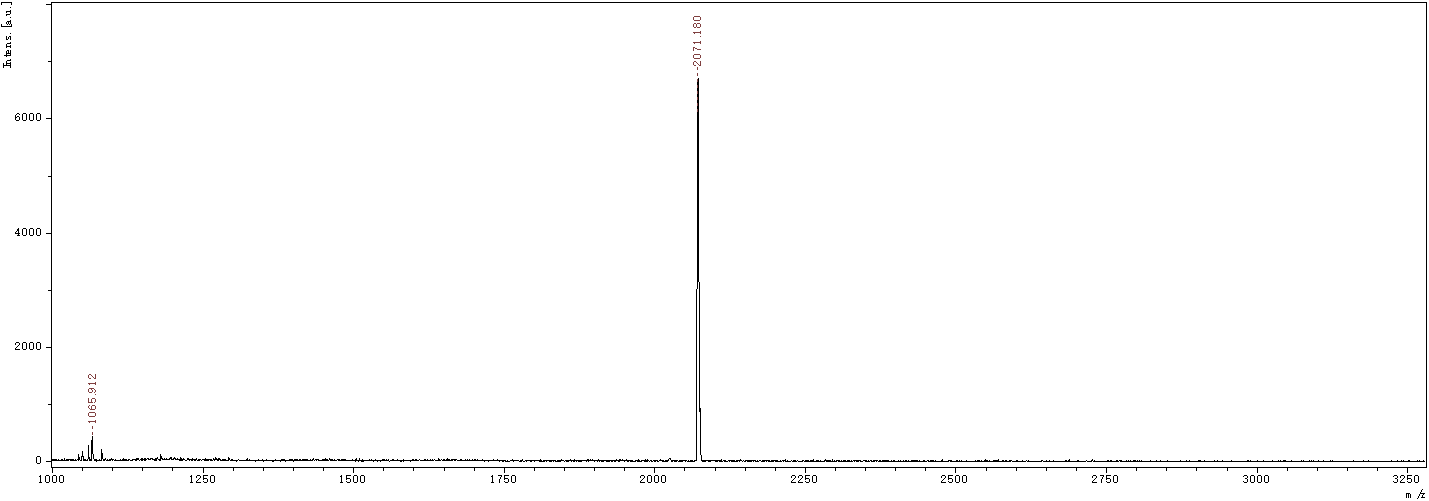


**Figure S18**. MS image of **I-QTP-4F**.

**Device fabrication and characterization**

**OSCs fabrication:** The patterned indium tin oxide (ITO, sheet resistance = 15 Ω square^-1^) glass substrates were sequentially ultrasonicated with detergent, deionized water, acetone, and isopropanol. Then, the ITO glasses were treated with UV-ozone for 20 min. Poly(3,4-ethylenedioxythiophene):polystyrene sulfonate (PEDOT:PSS) (Bay PVP. Al 4083, Bayer AG) was filtrated through a 0.45 *μ*m nylon filter and then spin-coated on the cleaned ITO substrates at 5000 rpm for 60 s to form a thin layer (30 nm). The PM6:L8-BO:acceptor (H-QTP-4F, Cl-QTP-4F, Br-QTP-4F and I-QTP-4F) (1:1:0.2, weight ratio of donor:acceptor_1_:acceptor_2_) blends were dissolved in chloroform (CF) with the solvent additive of diiodomethane (DIM) (0.4%, v/v) under 50 ℃ for 2 hours to mix intensively in a *N*_2_-filled glove box. The concentration is 16.5 mg m^1^ in total of PM6:L8-BO:acceptor. The blend solutions were spin-coated on the top of ITO/PEDOT:PSS substrates at a speed of 3000 rpm for 30 s, followed by a 85 ℃ for 6 min thermal annealing. The PDIN solution (2.0 mg ml^-1^ in methanol with 0.3 vol% acetic acid) was spin-coated on the top of active layers at 5000 RPM for 30 s. Finally, argentum electrode (Ag, 100 nm) was deposited under high vacuum (~10^-5^ Pa) in an evaporation chamber. The device area was exactly fixed at 0.038 mm^2^.

**OSCs measurement**: The current-voltage (*J-V*) curves were measured on a computer controlled Keithley 2400 Source under AM 1.5G (100 mW cm^-2^) using a solar simulator (XES-70S1, SAN-EI), which was calibrated by a standard Si solar cell (AK-200, Konica Minolta, Inc.). The light intensity was determined by a 2 × 2 cm^2^ standardized mono silicon cell (Oriel PN 91150V, Newport, USA.) calibrated by the National Renewable Energy Laboratory (NREL). The EQE values were measured with an EQ-R solar quantum efficiency test system (Enlitech Co., Ltd., Taiwan, China). All fabrication and characterization processes, except for the hole transport layers (HTLs) preparation and EQE measurements, were conducted in a high purity argon filled glove box. Transient photovoltage (TPV), transient photocurrent (TPC), and photo-induce charge extraction linear increasing voltage (Photo-CELIV) were conducted with the Paioscarrier measurement system (FLUXiM AG, Switzerland). A high-power white LED is utilized as light source for TPV, TPC and photo-CELIV measurements. The integrated power of the LED is 72 mW cm^-2^, and the spectrum distribution is mainly in the wavelength range of 440-470 nm and 540-630 nm, and the peak value located at 460 nm and 550 nm. Fourier transform photocurrent spectroscopy (FTPS) was measured using an integrated system (PECT-600, Enlitech), where the photocurrent was amplified and modulated by a lock-in instrument. Electroluminescence external quantum efficiency (EQE_EL_) was performed by applying external voltage/current sources through the devices (ELCT-3010, Enlitech). Femtosecond-resolved transient absorption spectra (fs-TAS): The laser beam is supplied by amplified Ti: sapphire laser source (800 nm, Coherent) that provides 100 fs pulses with a repetition rate of 1 kHz. The output was split into two beams, the stronger one of which was used (or frequency doubled to 400 nm) as the pump light, and the other one was focused into a sapphire plate to generate a broadband probe white light. Using an optical chopper, the repetition rate of the pump pulses was adjusted to 500 Hz, and were focused on the sample with the probe pulse (white light). The TA spectra were obtained by comparing the probe light spectra with and without pump light excitation. The photoinduced absorption change as a function of wavelength was described using optical density (absorbance) changes (*Δ*OD(*λ*)). By adjusting the delay time between the pump and probe pulses, a 3D transient spectral image *Δ*OD(*λ*, t) was formed. The intensity of the pump laser fluence was estimated to be 0.7 µJ cm^-2^ at 800 nm excited light.

**Charge mobility measurement by SCLC method:** The structure of electron-only devices is ITO/ZnO/active layer/PDIN/Al and the structure of hole-only devices is ITO/PEDOT:PSS/active layer/MoO_3_/Ag.^4^ The fabrication conditions of the active layer films are same with those for the OSCs. The charge mobilities are generally described by the Mott-Gurney equation (1):

$J=\frac{9}{8}\varepsilon_{r}\varepsilon_{0}\mu\frac{V^{2}}{L^{3}}$ (1)

where *J* is the current density, *ε_0_* is the permittivity of free space (8.85×10^-14^ F/cm), *ε_r_* is the dielectric constant of used materials, *μ* is the charge mobility, *V* is the applied voltage and L is the active layer thickness. The *ε_r_* parameter is assumed to be 3, which is a typical value for organic materials. In organic materials, charge mobility is usually field dependent and can be described by the disorder formalism, typically varying with electric field, E=V/L, according to the equation (2):

$\mu=\mu_{0}\exp[0.89\gamma\sqrt{\frac{V}{L}}]$ (2)

where *μ_0_* is the charge mobility at zero electric field and *γ* is a constant. Then, the Mott-Gurney equation can be described by (3):

$J=\frac{9}{8}\varepsilon_{r}\varepsilon_{0}\mu_{0}\frac{V^{2}}{L^{3}}\exp[0.89\gamma\sqrt{\frac{V}{L}}]$ (3)

In this case, the charge mobilities were estimated using the following equation (4):

$\ln(\frac{JL^{3}}{V^{2}})=0.89\gamma\sqrt{\frac{V}{L}}+\ln(\frac{9}{8}\varepsilon_{r}\varepsilon_{0}\mu_{0})$ (4)


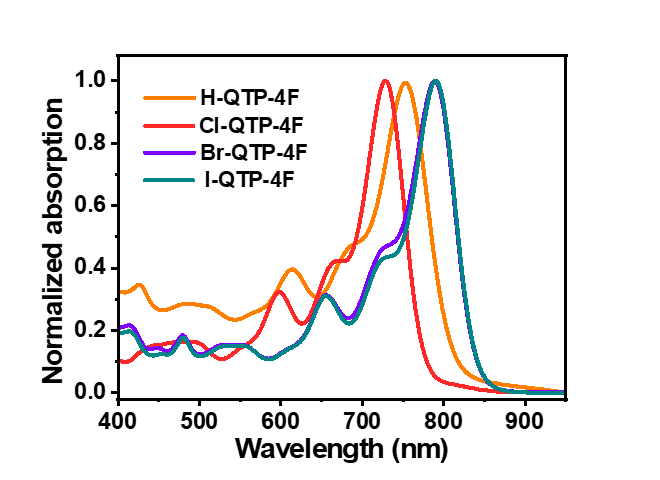


**Figure S19**. UV-vis absorption spectra of X-QTP-4F in chloroform.


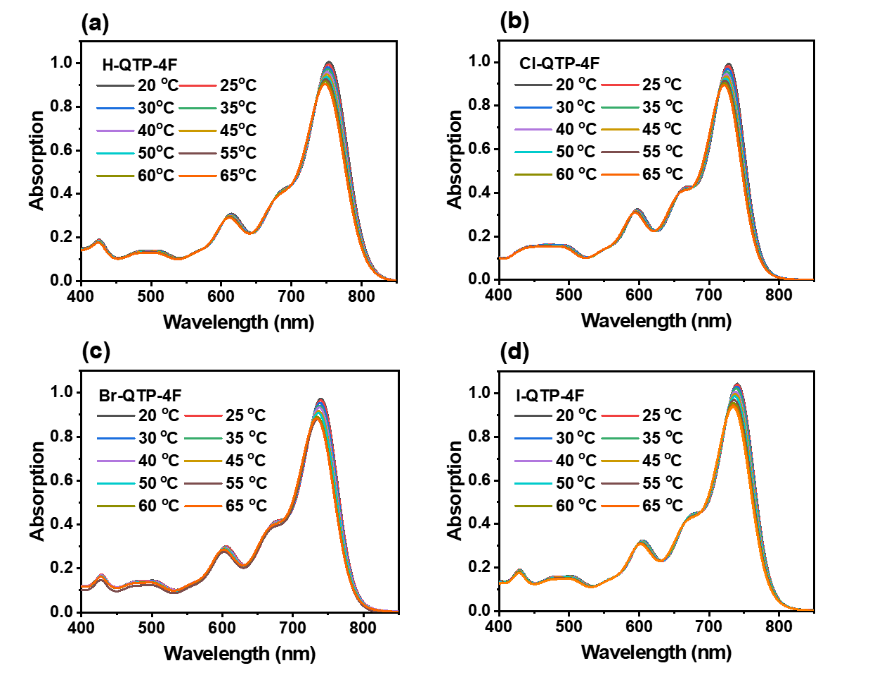


**Figure S20**. Temperature-dependent UV-vis absorption spectra of X-QTP-4F in chloroform.


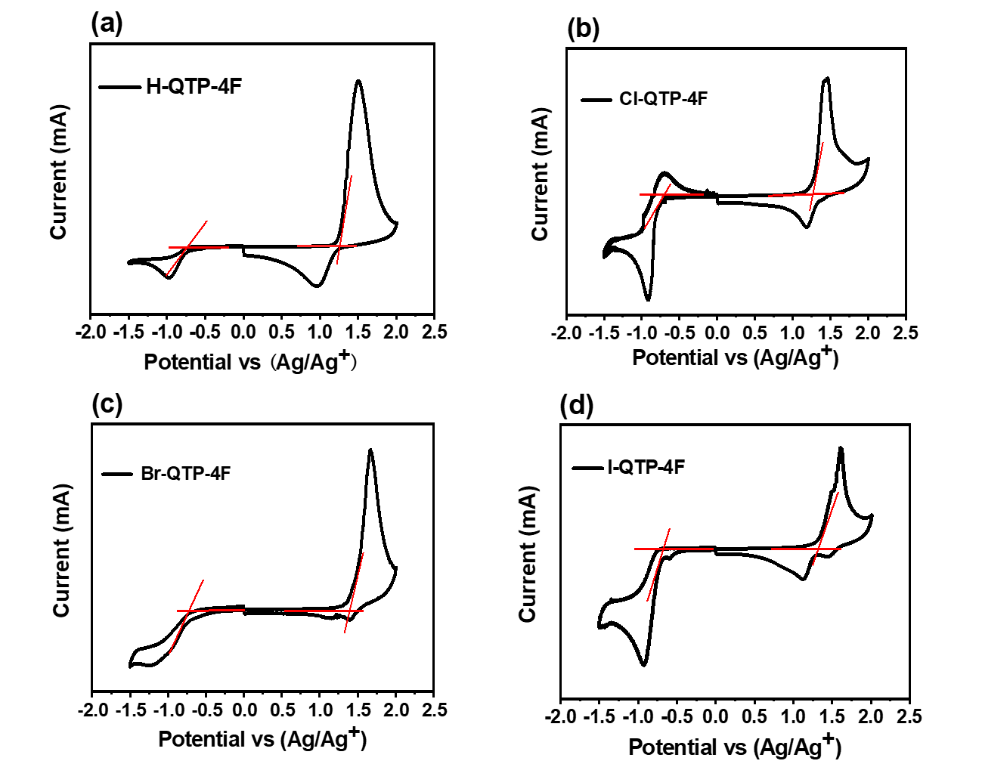


**Figure S21**. Cyclic voltammograms of X-QTP-4F in drop-cast thin films.

| (a) H-QTP-4F | |
| --- | --- |
| Top view  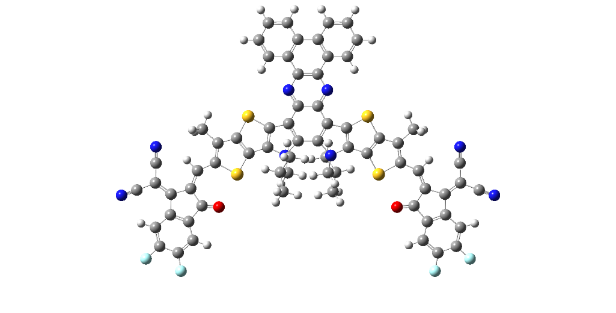 | LUMO: -3.48 eV  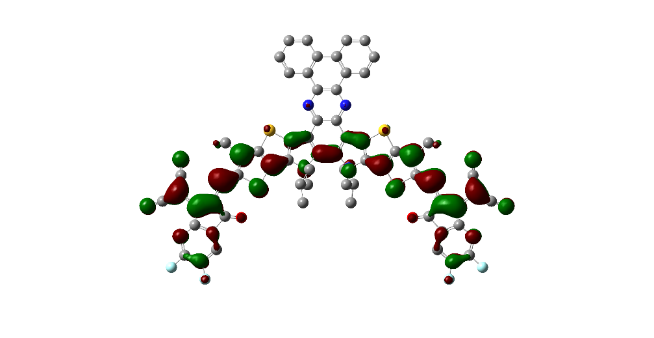 |
| Side view  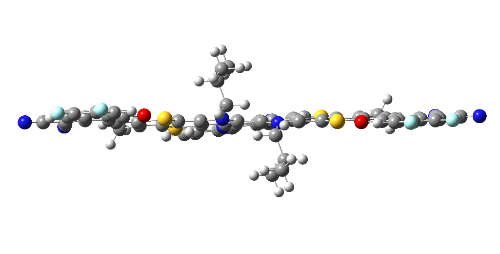 | HOMO: -5.50 eV  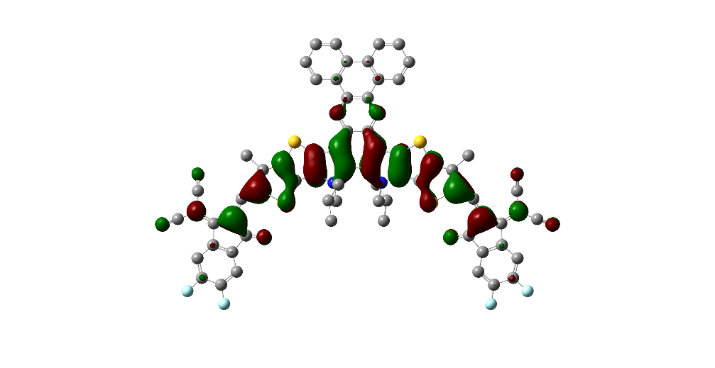 |
| (b) Cl-QTP-4F | |
| Top view  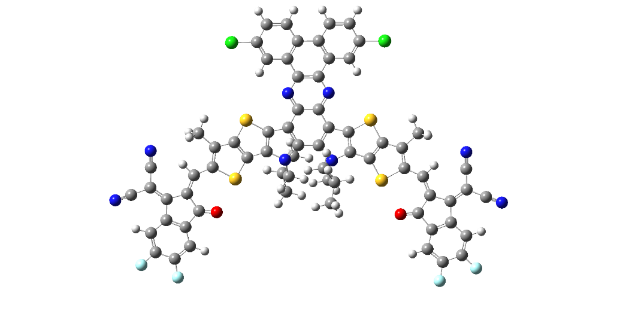 | LUMO: -3.52 eV  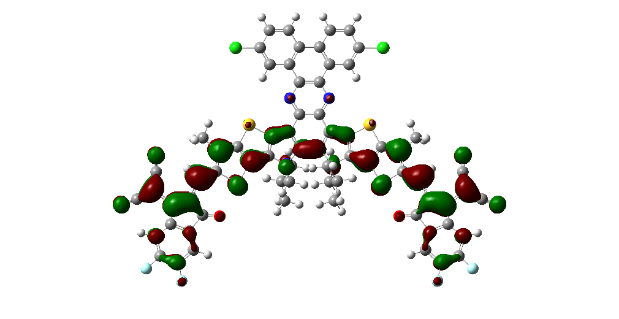 |
| Side view  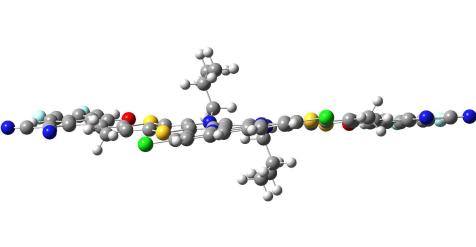 | HOMO: -5.57 eV  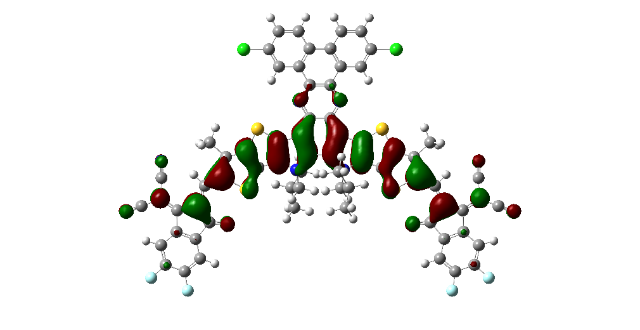 |
| (c) Br-QTP-4F | |
| Top view  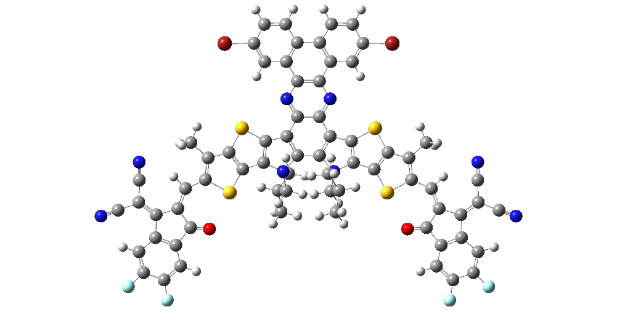 | LUMO: -3.52 eV  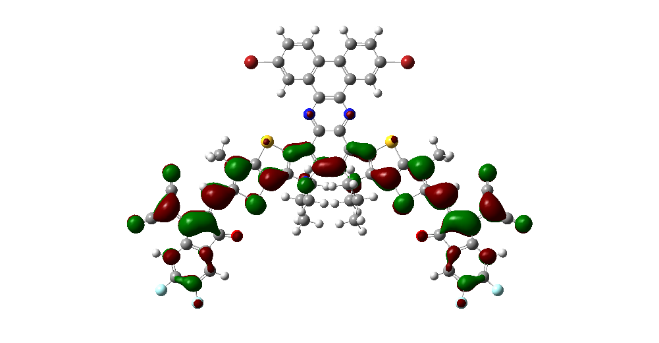 |
| Side view  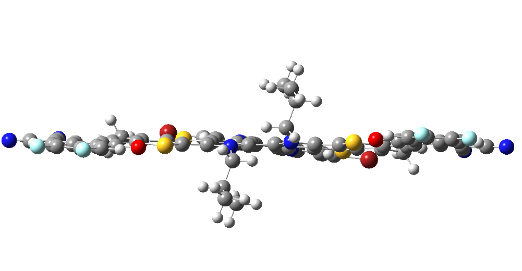 | HOMO: -5.57 eV  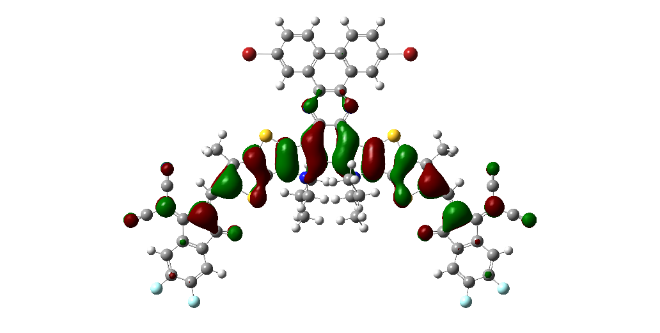 |
| (d) I-QTP-4F | |
| Top view  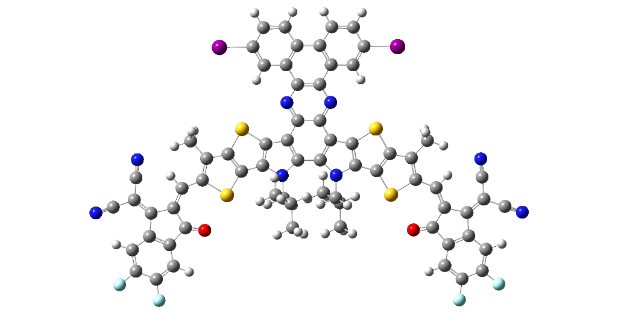 | LUMO: -3.54 eV  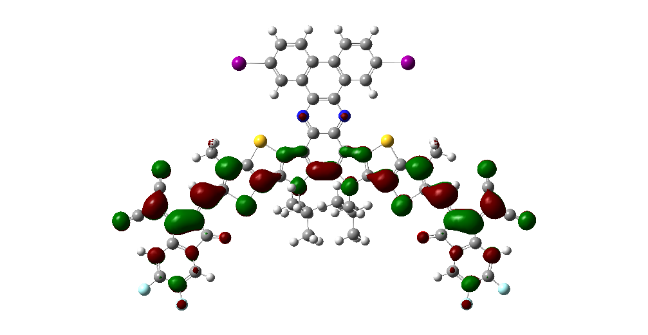 |
| Side view  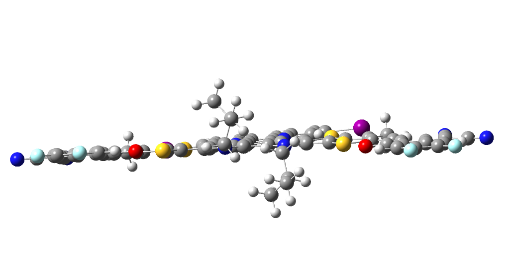 | HOMO: -5.60 eV  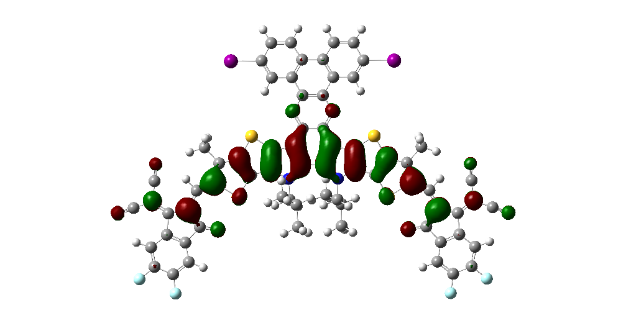 |

**Figure S22.** Calculated molecular geometries and frontier molecular orbitals of (a) H-QTP-4F, (b) Cl-QTP-4F, (c) Br-QTP-4F and (d) I-QTP-4F at B3LYP/6-31G(d,p) level.

**Figure S23.** super cells of X-QTP-4F (Version 2).

**Figure S24.** super cells of X-QTP-4F (Version 3).

**Figure S25.** The measurements of *T*_g_ values by fitting UV-vis deviation metric results.

**Figure S26**. The ln(JL^3^ /V^2^ ) vs (V/L)^0.5^ curves of (a) electron-only devices with the structure of ITO/ZnO/active layer/PDIN/Ag and (b) hole-only devices with the structure of ITO/PEDOT:PSS/active layer/MoO_3_/Ag.

**Figure S27**. 2D GIWAXS patterns for the pure film of X-QTP-4F.

**Figure S28**. (a) 2D GIWAXS patterns for the PM6:L8-BO blend film, (b) the corresponding line-cut profiles from GIWAXS data of PM6:L8-BO blend film.

**Figure S29**. AFM (a) height, (b) phase images and (c) the line profiles from AFM phase images (labeled the white line) to obtain the FWHM of cross-section for the PM6:L8-BO blend films.^5^


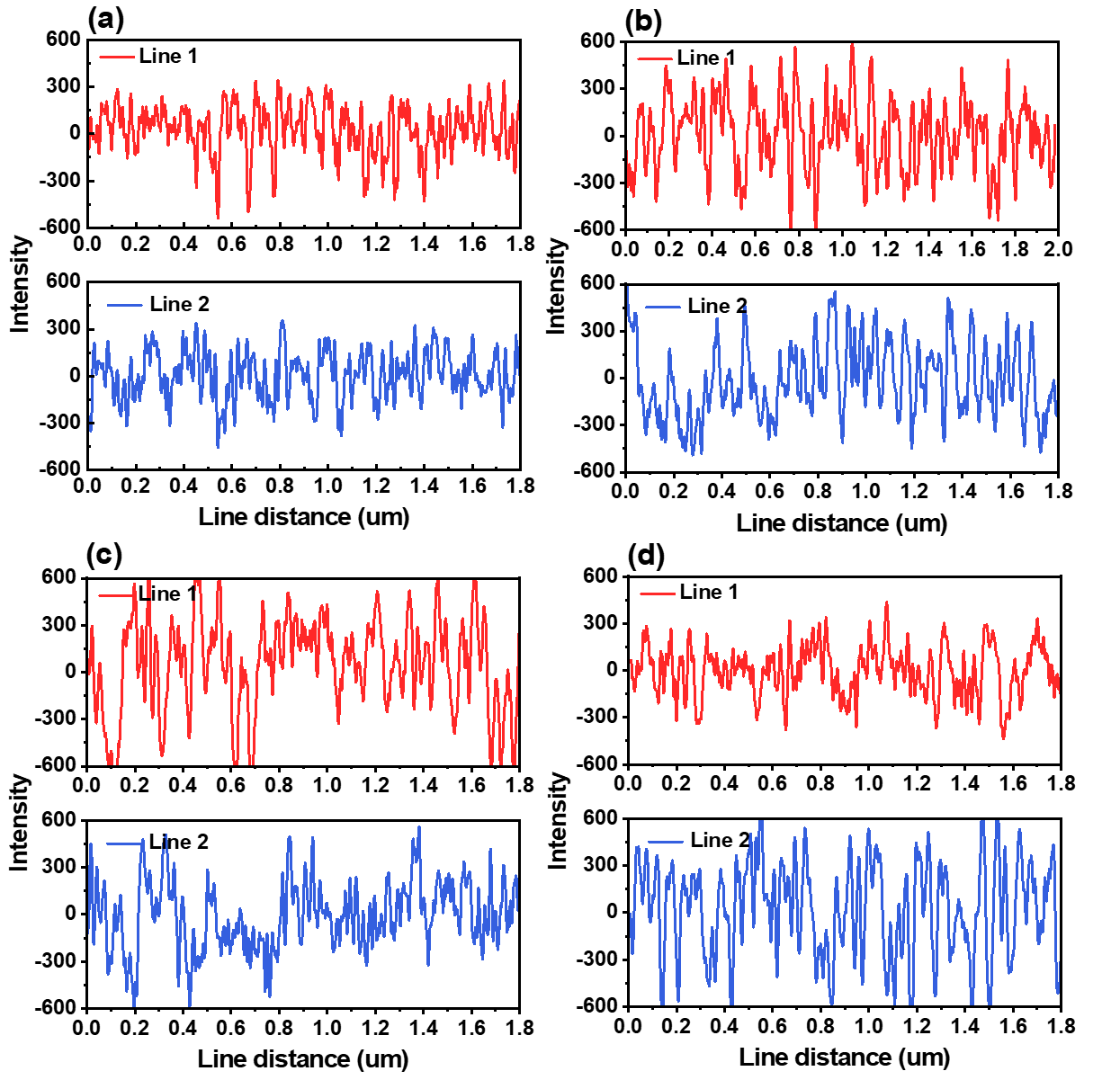


**Figure S30**. The line profiles from AFM phase images (labeled the white line) to obtain the FWHM of cross-section for the blend films of (a) PM6:L8-BO:H-QTP-4F, (b) PM6:L8-BO:Cl-QTP-4F, (c) PM6:L8-BO:Br-QTP-4F and (d) PM6:L8-BO:I-QTP-4F.


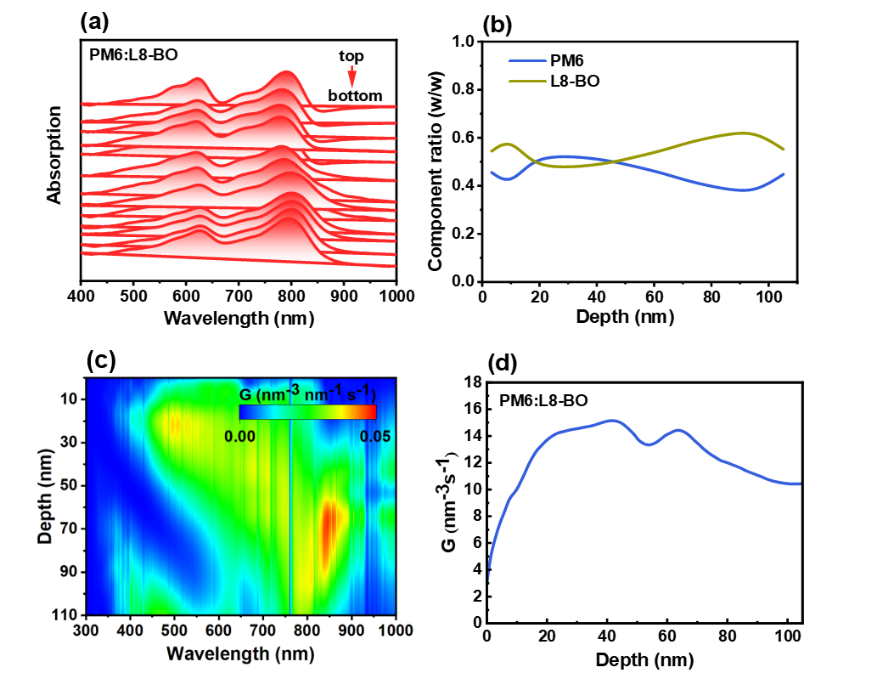


**Figure S31.** (a) FLAS of blend film for PM6:L8-BO. (b) Film-depth-dependent PM6, and L8-BO distribution. (c) Exciton generation contours as numerically simulated from the FLAS for PM6:L8-BO. (d) Dependence of the simulated exciton generation rate (G) on the film depth of blend film for PM6:L8-BO.


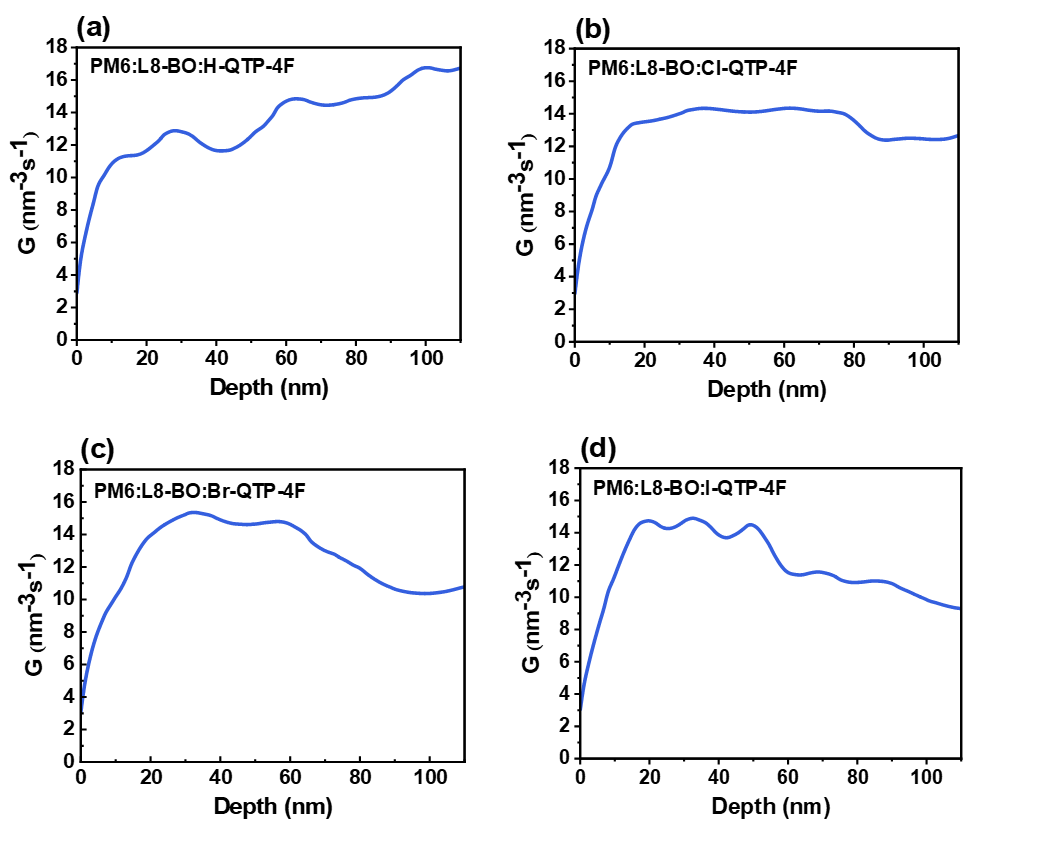


**Figure S32.** (a-d) Dependence of the simulated exciton generation rate (G) on the film depth of blend film for PM6:L8-BO:X-QTP-4F.


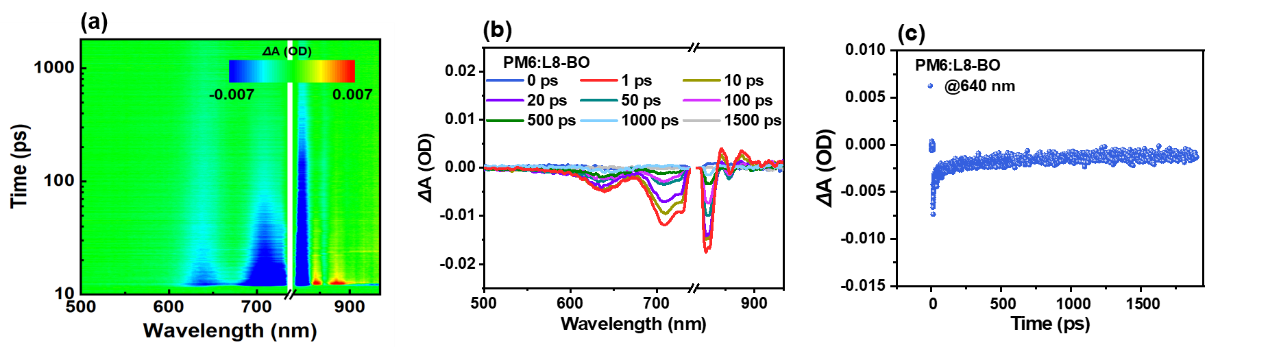


**Figure S33.** (a) TA spectra of blend film for PM6:L8-BO under 800 nm laser pumping. (b) TA spectra of blend film for PM6:L8-BO at different delay times. (i) Kinetic traces of blend film for PM6:L8-BO at the selected wavelength.

**References**

1. Y. Wang, J. Xue, H. Zhong, C.R. Everett, X. Jiang, M.A. Reus, A. Chumakov, S.V. Roth, M.A. Adedeji, N. Jili, K. Zhou, G. Lu, Z. Tang, G.T. Mola, P. Müller-Buschbaum, W. Ma, *Adv. Energy Mater.* **2023**, *13*, 2203496.

2. H. Tian, W. Xu, Z. Liu, Y. Xie, W. Zhang, Y. Xu, S. Y. Jeong, F. Zhang, N. Weng, Z. Zhang, K. Wang, Q. Sun, J. Zhang, X. Li, X. Du, X. Hao, H. Y. Woo, X. Ma, F. Zhang, *Adv. Funct. Mater.* **2024**, *34*, 2313751.

3. C. Li, J. Zhou, J. Song, J. Xu, H. Zhang, X. Zhang, J. Guo, L. Zhu, D. Wei, G. Han, J. Min, Y. Zhang, Z. Xie, Y. Yi, H. Yan, F. Gao, F. Liu, Y. Sun, *Nat. Energy*, **2021**, 6, 605-613.

4. C. Yang, M. Jiang, S. Wang, B. Zhang, P. Mao, H. Y. Woo, F. Zhang, J. Wang, Q. An, Hot-Casting Strategy Empowers High-Boiling Solvent-Processed Organic Solar Cells with Over 18.5% Efficiency. *Adv. Mater.* **2024**, *36*, 2305356.

5. L. Zhu, M. Zhang, J. Xu, C. Li, J. Yan, G. Zhou, W. Zhong, T. Hao, J. Song, X. Xue, Z. Zhou, R. Zeng, H. Zhu, C.-C. Chen, R.C.I. MacKenzie, Y. Zou, J. Nelson, Y. Zhang, Y. Sun, F. Liu, *Nature Mater*. **2022**, *21*, 656.
